# Comparative evaluation of left atrial appendage occlusion and oral anticoagulation: a Bayesian meta-analysis of randomized controlled trials

**DOI:** 10.1093/europace/euag163

**Published:** 2026-07-23

**Authors:** Krishna Saketh Athmakuri, Shreyas Raghavan Nandyal, Hrushikesh Reddy Pamreddy, Saketh Vinjamuri, Shanmukh S P Lingamsetty, Anushka Vishwas Mahajan, Muqaddas Hussain, Hari P Chaliki, Komandoor Srivathsan

**Affiliations:** Department of Cardiovascular Medicine, Mayo Clinic Arizona, 5777 East Mayo Blvd. Phoenix, AZ 85054, USA; Department of Internal Medicine, John H Stroger Jr. Hospital of Cook County, Chicago, IL, USA; Department of Internal Medicine, Saint Vincent Hospital, Worcester, MA, USA; Department of Internal Medicine, Cleveland Clinic Fairview Hospital, Cleveland, OH, USA; Division of Medicine, Beth Israel Deaconess Medical Center, Boston, MA, USA; Department of Cardiovascular Medicine, Mayo Clinic Arizona, 5777 East Mayo Blvd. Phoenix, AZ 85054, USA; Department of Cardiovascular Medicine, Mayo Clinic Arizona, 5777 East Mayo Blvd. Phoenix, AZ 85054, USA; Department of Cardiovascular Medicine, Mayo Clinic Arizona, 5777 East Mayo Blvd. Phoenix, AZ 85054, USA; Department of Cardiovascular Medicine, Mayo Clinic Arizona, 5777 East Mayo Blvd. Phoenix, AZ 85054, USA

**Keywords:** Left atrial appendage occlusion, Oral anticoagulation, Atrial fibrillation, Bayesian meta-analysis, Stroke prevention, Bleeding

## Abstract

**Aims:**

Randomized trials comparing percutaneous left atrial appendage occlusion (LAAO) with oral anticoagulation (OAC) for stroke prevention in patients with atrial fibrillation have yielded conflicting results across device generations and anticoagulant eras. This Bayesian meta-analysis of randomized trials aimed to compare LAAO vs. OAC for stroke or systemic embolism (SSE) and non-procedural clinically relevant bleeding (CRB).

**Methods and results:**

We conducted a systematic search across five databases (PubMed, Embase, Scopus, Web of Science, and Cochrane CENTRAL) to identify randomized trials of LAAO vs. OAC. Primary outcomes of stroke or systemic embolism, non-procedural CRB and clinical benefit composite were estimated using a Bayesian hierarchical random effects model and findings confirmed across robust sensitivity and subgroup analyses. Posterior probabilities for superiority, non-inferiority and equivalence were estimated using clinically meaningful margins. Six randomized trials including 7004 participants in the intention-to-treat groups, were analysed. LAAO was not associated with lower SSE vs. OAC (pooled RR, 1.11; 95% CrI, 0.80–1.49), with a 74.6% posterior probability of favouring OAC. The posterior probability of crossing non-inferiority was P(HR < 1.4) of 89%. In contrast, LAAO reduced non-procedural CRB (pooled RR, 0.59; 95% CrI, 0.45–0.78). The net clinical benefit composite, pooling contemporary trials, demonstrated a pooled RR of 0.90 (95% CrI 0.51–1.50), with P(RR < 1) of 76%, with substantial heterogeneity for this outcome.

**Conclusion:**

This Bayesian meta-analysis found no clear evidence supporting clinically meaningful non-inferiority of LAAO over OAC for stroke prevention. Despite reduced non-procedural bleeding, LAAO did not demonstrate a similar reduction in major bleeding.

## Graphical Abstract

**Graphical Abstract:**
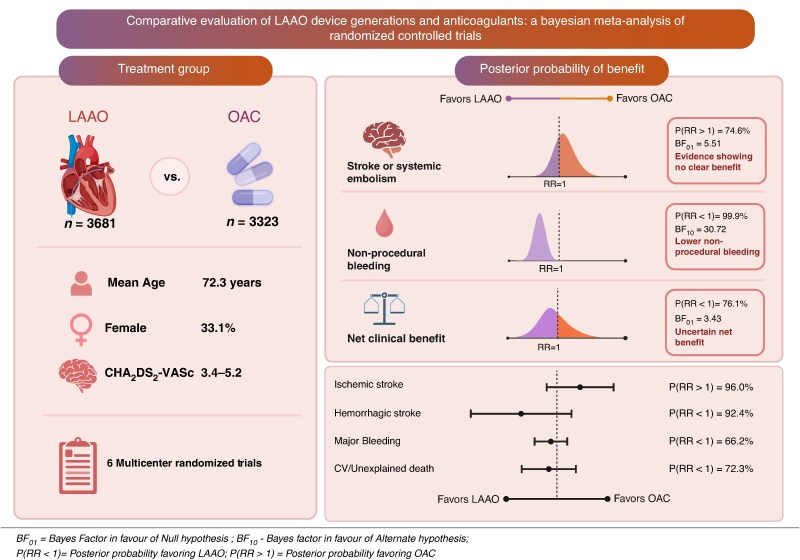
*Created in BioRender. saketh, K. (2026)*  https://BioRender.com/y0lri6f. Comparative Evaluation of Left Atrial Appendage Occlusion And Oral Anticoagulation: A Bayesian Meta-Analysis of Randomized Controlled Trials. Shaded areas in purple represent probability of benefit with LAAO. LAAO shows high probability of reducing non-procedural bleeding but trails behind in efficacy for long-term stroke prevention. BF, Bayes factors; LAAO, left atrial appendage occlusion; OAC, oral anticoagulation; RR, risk ratio.

What’s New?

- We used a Bayesian framework to quantify posterior probability for superiority, non-inferiority, and equivalence of LAAO vs. OAC across multiple patient-important outcomes.
- LAAO did not demonstrate a reduction in stroke or systemic embolism, with a 74.6% posterior probability of favouring OAC and an 89% probability of non-inferiority using a conventional non-inferiority margin.
- Subgroup analyses restricted to contemporary direct oral anticoagulant-era and newer-generation device trials yielded results consistent with the primary analysis, suggesting the bleeding advantage of LAAO is durable while the stroke benefit trails behind standard therapy.

## Introduction

Atrial fibrillation (AF) is the most common sustained cardiac arrhythmia encountered in clinical practice, affecting more than 60 million individuals worldwide.^1,2^ Patients with AF face an elevated risk of stroke and systemic embolism.^3^ Oral anticoagulation (OAC) historically with vitamin K antagonists and now predominantly with direct oral anticoagulants (DOACs), remains the standard of care for stroke prevention in patients with elevated CHA_2_DS_2_-VASc scores.^4^ However, long-term anticoagulation carries a risk of major and clinically relevant non-major bleeding, is constrained by suboptimal adherence, drug interactions, and dose adjustments, and requires indefinite continuation in most patients.^5,6^

The left atrial appendage (LAA) has long been thought to be the origin of approximately 90% of cardiogenic thrombi in non-valvular AF, providing the anatomical rationale for catheter-based left atrial appendage occlusion (LAAO) as alternative to lifelong OAC.^7,8^ LAAO has principally been offered to patients who cannot tolerate OAC, especially those at elevated bleeding risk, with contraindications to OAC, or in whom adherence is uncertain.^9,10^ The device landscape has evolved considerably: the first-generation Watchman received FDA approval in 2015, followed by the iteratively redesigned Watchman FLX in 2020, and the Amplatzer Amulet has expanded the available platform options.^11,12^

The randomized evidence base for LAAO has grown substantially over the past decade, yet the accumulated data present a conflicting picture. The PROTECT AF and PREVAIL trials established non-inferiority of the first-generation Watchman against warfarin, with extended follow-up suggesting superiority for haemorrhagic stroke and all-cause mortality.^13^ PRAGUE-17 demonstrated non-inferiority of LAAO against OACs, and OPTION and CHAMPION-AF evaluated the newer Watchman FLX against contemporary DOAC therapy in larger cohorts.^14–16^ However, CLOSURE-AF, the longest-duration trial to date, reported that LAAO failed to meet non-inferiority for its primary efficacy endpoint and raised concern about increased stroke risk and mortality in the device arm.^17^ These conflicting findings track with device generation: older trials evaluated older devices against warfarin, while newer trials have tested next-generation platforms against DOACs, making it difficult to draw unified conclusions across the evidence base.

Prior meta-analyses have attempted to reconcile these discrepancies using conventional frequentist methods.^18–20^ However, frequentist frameworks carry inherent limitations in this context: they cannot generate direct probability statements about treatment effects, and are susceptible to repeated-testing inflation as trials accumulate.^21,22^

A Bayesian meta-analytic framework addresses these limitations directly. Bayesian inference yields probability distributions that enable clinically interpretable statements, such as the probability that LAAO is superior, inferior, or equivalent to OAC for a given endpoint.^23^

The primary objective of this study was therefore to compare the effects of LAAO and OAC on stroke or systemic embolism and non-procedural CRB and other patient-important outcomes by applying a Bayesian random-effects model to all available randomized trial data.

## Methods

### Protocol and registration

This systematic review and meta-analysis was registered with PROSPERO (ID: [CRD420251266412]) and was reported in accordance with the Preferred Reporting Items for Systematic Reviews and Meta-Analyses (PRISMA 2020) checklist (see Supplementary Supplementary material online, appendix methods  *S1* and *S2*). Ethical approval was not required as this study analysed previously published data.

### Eligibility criteria

Studies were eligible for inclusion if they (1) were randomized controlled trials (RCTs); (2) enrolled adults (≥18 years) with non-valvular AF (3) compared percutaneous, catheter-based left atrial appendage occlusion (LAAO) vs. best medical therapy with either vitamin K antagonists (VKA) or DOAC; and (4) presented data on outcomes of interest, including stroke or systemic embolism, non-procedural CRB, ischaemic stroke, haemorrhagic stroke, and cardiovascular (CV) mortality.

Exclusion criteria were as follows: (1) non-original studies; (2) single-arm studies (e.g. AMULET IDE, PINNACLE FLX), observational designs, crossover trials, and subgroup or post-hoc analyses of RCTs; (3) trials comparing antithrombotic regimens after LAAO (e.g. ANDES); and (4) trials of surgical LAAO or incidental LAAO during concomitant cardiac surgery (e.g. LAAOS III).

When multiple follow-up periods were available, the longest was selected (e.g. 5-year PROTECT AF and PREVAIL outcomes; 4-year PRAGUE-17 outcomes). All analyses used intention-to-treat (ITT) populations.

### Information sources

A systematic search was conducted across five databases: PubMed, Embase (via Ovid), Scopus, Web of Science, Cochrane Central Register of Controlled Trials (CENTRAL), and trial registries: ClinicalTrials.gov. Only publications in English were eligible for screening. The trial protocols of CLOSURE AF and CHAMPION AF trials were included after initial screening and their results, were included after their publication. The complete search strategy is presented in Supplementary Supplementary material online, Appendix  *S3*.

### Study selection and data extraction

Retrieved records were de-duplicated prior to screening. Title and abstract screening, followed by full-text review of potentially eligible records, were performed independently by two authors; discordant assessments were resolved through discussion with a senior author. Reasons for exclusion at the full-text stage are documented in the Supplementary Supplementary material online, Appendix  *S4*. Data extraction was performed independently by the two authors, capturing trial-level characteristics, follow-up duration, device type, anticoagulation regimen, outcome-level event counts, and adjusted effect estimates.

### Outcomes

#### Primary and secondary outcomes

Co-primary outcomes were stroke or systemic embolism (SSE) and non-procedural CRB. SSE encompassed ischaemic stroke, haemorrhagic stroke, and peripheral systemic embolism, and was directly comparable across all trials. Trial-reported HRs for stroke, systemic embolism, or all-cause stroke was also analyzed. Clinically relevant bleeding (CRB) in this context represents a composite of major and non-major bleeding events. Non-procedural CRB was defined as CRB occurring outside each trial's peri-procedural window. Operationalized outcome definitions, including trial-specific definitions, are presented in Supplementary Supplementary material online, Appendix Methods  *S5*. Pre-specified secondary outcomes included clinically meaningful endpoints of ischaemic stroke, haemorrhagic stroke, CV or unexplained death, and major bleeding (procedural and non-procedural bleeding). Adjudicated event counts at longest available follow-up were extracted and analyzed using the same Bayesian framework.

#### Clinical benefit composite

A clinical benefit composite was constructed to capture the net effect of LAAO by integrating efficacy and safety components. Three trials contributed directly usable composites: the CLOSURE-AF and PRAGUE-17 primary efficacy endpoints, and the CHAMPION-AF net clinical benefit endpoint. PROTECT AF, PREVAIL, and OPTION were not included in this pooling as their pre-specified composites did not include a bleeding component.

#### Quality assessment

Risk of bias was assessed at the outcome level for the co-primary outcomes using the Cochrane Risk of Bias 2 (RoB2) tool. Five domains were evaluated for each trial: (D1) bias arising from the randomization process, (D2) bias due to deviations from intended interventions, (D3) bias due to missing outcome data, (D4) bias in measurement of the outcome, and (D5) bias in selection of the reported result. Each domain was classified as low risk of bias, some concerns, or high risk.^24^

#### Statistical analysis

A Bayesian random-effects meta-analysis was conducted for each outcome using a normal-normal hierarchical model, accounting for within-study uncertainty and between-study heterogeneity. Model convergence was confirmed using standard diagnostic criteria. For primary analysis, a weakly informative prior was placed on the pooled effect estimate and between-study heterogeneity parameters. The prior specification for primary analysis was a normal distribution centred on median of null effect (RR or HR of 1) and a standard deviation of 1, allowing realistic values of these ratio measures to drive posteriors. These parameters are expressed as median value along with their 95% credible interval (CrI), a range within which the true effect lies with 95% probability. Bayes factors were computed for each outcome to quantify the evidence for or against treatment effect, with the null hypothesis of no difference (BF_01_) vs. the alternative hypothesis (BF_10_) of a true difference and interpreted according to conventional scales of increasing integer values. In addition to these, we also report the posterior probability of non-inferiority for primary efficacy outcome, using a margin of 1.4 and a tighter margin of 1.25 for these ratio measures.^17^

Six prespecified sensitivity analyses assessed robustness to alternative priors, including priors that vary in heterogeneity weight, skepticism about the magnitude of the treatment effect, and informative priors from the medical literature. A final analysis incorporated informative priors for SSE and CRB derived from a published meta-analysis of PROTECT AF, PREVAIL, PRAGUE-17, and OPTION, formally updating them with additional data from CLOSURE-AF and CHAMPION-AF.

To evaluate the influence of older warfarin-era trials and device generation, two subgroup analyses were performed. The first excluded PROTECT AF and PREVAIL, retaining contemporary trials with substantial DOAC use (control-based subgroup). The second excluded PRAGUE-17, restricting analysis to trials using newer-generation devices and contemporary DOAC comparators (device-based subgroup). All analyses were conducted in R using the brms, Stan, meta, metafor, tidybayes, and bayesplot packages. Figures were generated with ggplot2 and ggridges.

## Results

**Figure 1:**
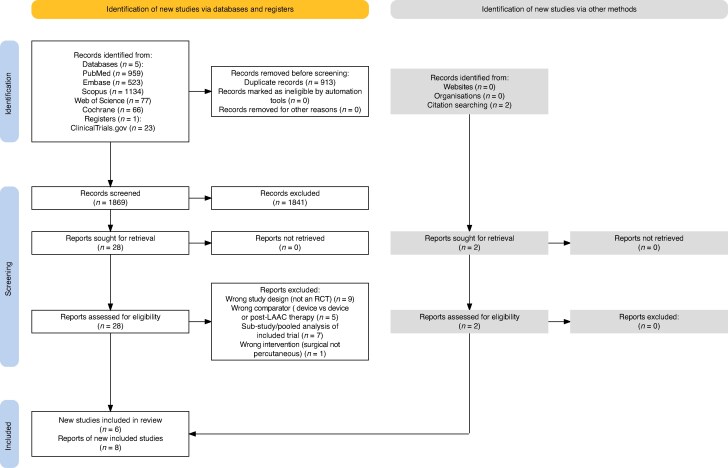
PRISMA flow diagram. PRISMA 2020 flow diagram showing inclusion of randomized trials comparing left atrial appendage occlusion with oral anticoagulation.

The results of the search strategy are summarized in *Figure 1*. The final dataset comprised five trial-level units of analysis corresponding to six randomized trials. The six trials enrolled 7004 participants in the ITT group with Mean age ranging from 70 to 78 years, the proportion of women from 30% to 39%, mean CHA_2_DS_2_-VASc scores from 3.4 to 5.2, and mean HAS-BLED scores from 1.2 to 3.1. Further trial-level characteristics are summarized in *Table 1*.

**Table 1:** Baseline characteristics of patients included in randomized trials of left atrial appendage occlusion vs. anticoagulation

Characteristic	PROTECT AF		PREVAIL		PRAGUE-17		OPTION		CLOSURE-AF		CHAMPION-AF	
	Device	Control	Device	Control	Device	Control	Device	Control	Device	Control	Device	Control
Trial arm	Watchman	Warfarin	Watchman	Warfarin	Watchman/Amulet	DOACs	Watchman FLX	OAC	Watchman/Amulet	Best medical care	Watchman FLX	NOACs
Age, years	71.7 ± 8.8	72.7 ± 9.2	74.0 ± 7.4	74.9 ± 7.2	73.4 ± 6.7	73.2 ± 7.2	69.7 ± 7.4	69.4 ± 7.9	78.5 ± 6.8	77.3 ± 7.3	71.6 ± 7.5	71.8 ± 7.5
Female sex, *n* (%)	137 (29.6)	73 (29.9)	87 (32.3)	35 (25.4)	67 (33.3)	71 (35.3)	283 (35.2)	263 (33.0)	172 (38.6)	171 (38.7)	485 (32.4)	473 (31.5)
CHA_2_DS_2_-VASc score	3.4 ± 1.5	3.7 ± 1.6	4.0 ± 1.2	4.1 ± 1.2	4.7 ± 1.5	4.7 ± 1.5	3.5 ± 1.3	3.5 ± 1.3	5.2 ± 1.5	5.1 ± 1.6	3.5 ± 1.2	3.5 ± 1.3
HAS-BLED score	NR^a^	NR^a^	NR^a^	NR^a^	3.1 ± 0.9	3.0 ± 0.9	1.2 ± 0.8	1.2 ± 0.8	3.1 ± 0.9	3.0 ± 0.9	1.3 ± 0.8	1.3 ± 0.8
AF type Paroxysmal, *n* (%)	200 (43.2)	99 (40.6)	131 (48.7)	71 (51.4)	53 (26.4)	67 (33.3)	477 (59.4)	501 (62.9)	NR	NR	1038 (69.3)	1028 (68.5)
AF type Persistent, *n* (%)	97 (21.0)	50 (20.5)	85 (31.6)	39 (28.3)	47 (23.4)	46 (22.9)	326 (40.6)	296 (37.1)	NR	NR	358 (23.9)	382 (25.4)
AF type Permanent, *n* (%)	160 (34.6)	93 (38.1)	42 (15.6)	22 (15.9)	83 (41.3)	72 (35.8)	NR	NR	NR	NR	102 (6.8)	91 (6.1)
Hypertension, *n* (%)	415 (89.6)	220 (90.2)	238 (88.5)	134 (97.1)	186 (92.5)	186 (92.5)	721 (89.8)	701 (88.0)	417 (93.5)	417 (94.3)	NR	NR
Diabetes mellitus, *n* (%)	113 (24.4)	72 (29.5)	91 (33.8)	41 (29.7)	73 (36.3)	90 (44.8)	222 (27.6)	221 (27.7)	175 (39.2)	186 (42.1)	NR	NR
Heart failure/CHF, *n* (%)	124 (26.8)	66 (27.0)	63 (23.4)	32 (23.2)	88 (43.8)	90 (44.8)	161 (20.0)	166 (20.8)	258/445 (58.0)	247/442 (55.9)	NR	NR
Prior stroke/TIA, *n* (%)	82 (17.7)	49 (20.1)	74 (27.5)	39 (28.3)	73 (36.3)	69 (34.3)	80 (10.0)	92 (11.5)	142/445 (31.9)	151/442 (34.2)	119 (7.9)	114 (7.6)
CAD/prior MI, *n* (%)	NR	NR	NR	NR	30 (14.9)	39 (19.4)	241 (30.4)	262 (33.1)	257/441 (58.3)	232/439 (52.8)	NR	NR
BMI/weight	BMI 31.6 ± 6.0	BMI 31.3 ± 6.2	NR	NR	86.9 ± 17.6 kg	88.1 ± 16.2 kg	BMI 30.7 ± 5.8	BMI 30.7 ± 6.1	BMI 27.6 ± 5.6	BMI 28.1 ± 6.9	NR	NR

Data are presented as mean ± SD or number (%). Characteristics are listed according to the device and control groups within each trial, as reported in the source publications. Because reporting differed across trials, some variables were unavailable for specific studies. AF, atrial fibrillation; BMI, body mass index; CAD, coronary artery disease; CHF, congestive heart failure; DOAC, direct oral anticoagulant; MI, myocardial infarction; NOAC, non-vitamin K antagonist oral anticoagulant; NR, not reported; OAC, oral anticoagulation; and TIA, transient ischaemic attack

^a^Percent of patients with HAS-BLED of 0, 1–2, >3 were 6.4% vs. 1.7%, 73.7% vs. 68.6%, 19.9% vs. 29.7% respectively.

### Quality assessment

For stroke or systemic embolism, all five trial-level assessments were rated as some concerns with none at high risk. For non-procedural CRB, four assessments had some concerns, and one was judged high risk, driven by heterogeneous major bleeding definition. Results are presented as outcome-specific traffic-light plots and summary bar plots (see Supplementary Supplementary material online, Appendix Methods  *S6*). Publication bias was assessed using funnel plots and Egger’s test (see Supplementary Supplementary material online, appendix  *S7*).

### Convergence diagnostics

All Bayesian models converged satisfactorily, with R-hat values ≤1.002 and bulk and tail effective sample sizes (ESS) well above the prespecified thresholds of R-hat <1.01 and ESS >400 (see Supplementary Supplementary material online, appendix Methods  *S8*).

### Stroke or systemic embolism (SSE)

**Figure 2:**
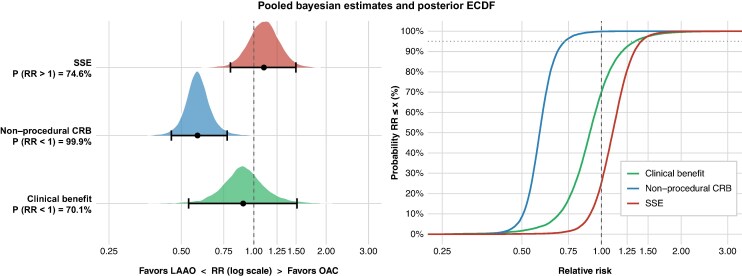
Bayesian posterior estimates across primary outcomes. The left panel shows posterior distributions of pooled RR for SSE, nonprocedural CRB, and clinical benefit; black dots indicate posterior medians and bars indicate 95% credible intervals. Right panel shows posterior empirical cumulative distribution functions. Dashed lines in both panels indicate RR of 1, i.e. Null effect. CRB, clinically relevant bleeding; ECDF, empirical cumulative distribution function; LAAO, left atrial appendage occlusion; RR, relative risk; SSE, stroke or systemic embolism.

Primary analysis using weakly informative priors yielded a pooled RR of 1.11 (95% CrI 0.801.49), with posterior probability favouring OAC P(RR > 1) = 0.746 (See *Figure 2*). The Bayes factor for this comparison was BF_01_ = 5.51. Between-study heterogeneity was low (τ = 0.18, 95% CrI 0.006–0.59). The pooled HR for this outcome, reported across four studies, was 1.11 (95% CrI 0.74–1.65), with P(HR > 1) = 0.729. On the HR scale for non-inferiority, P(HR < 1.4) was 89% and P(HR < 1.25) was 66%. On the RR scale, using a non-inferiority margin of RR < 1.25, the P(RR < 1.25) was 80.8%. Within the region of practical equivalence (ROPE) of 0.80 < RR < 1.25, the probability of equivalence P(0.80 < RR < 1.25) was 78.3%. Sensitivity analysis with informative priors derived from previous meta-analysis yielded a median RR of 1.04 (95% CrI 0.82–1.29) with P(RR > 1) of 64.3%. Comprehensive results of sensitivity analysis are reported in Supplementary Supplementary material online, Appendix Results  *S9*.

### Non-procedural CRB

For non-procedural CRB, the pooled RR was 0.59 (95% CrI 0.45–0.78), with P(RR < 1) = 0.999, Bayes factor BF_10_ = 30.72; however, between-study heterogeneity was modest (τ = 0.20, 95% CrI 0.010–0.58). The pooled HR for this outcome was 0.52 with 95% CrI (0.37–0.81), consistent with the expected true non-procedural bleeding reduction with LAAO.

### Clinical benefit composite

The clinical benefit composite was available for CLOSURE-AF, PRAGUE-17, and CHAMPION-AF. The pooled RR was 0.90 (95% CrI 0.51–1.50), with P(RR < 1) = 0.761 and BF_01_ = 3.432 (moderate evidence for null) with substantial between-study heterogeneity (τ = 0.41, 95% CrI 0.12–1.12).

### Secondary outcomes

**Figure 3:**
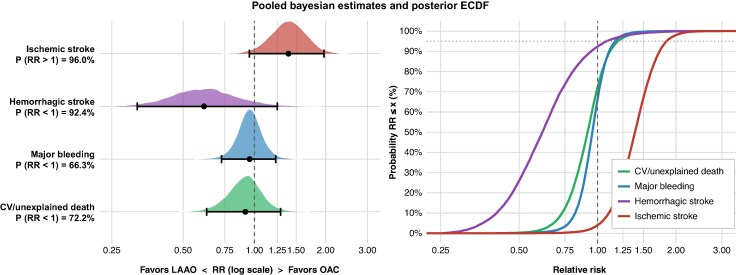
Bayesian posterior estimates for secondary outcomes. Left panel shows posterior distributions of pooled relative risks for ischaemic stroke, haemorrhagic stroke, major bleeding, and cardiovascular or unexplained death; dots indicate posterior medians and bars indicate 95% credible intervals. Right panel shows posterior empirical cumulative distribution functions, illustrating outcome-specific probabilities across RR thresholds. CV, cardiovascular; ECDF, empirical cumulative distribution function; LAAO, left atrial appendage occlusion; RR, relative risk.

Results for all secondary outcomes are presented in *Figure 3* and *Table 2*. LAAO was associated with numerically more ischaemic strokes (RR 1.41, 95% CrI 0.95–1.96), with the posterior probability favouring OAC of 95.5%. A numeric reduction in haemorrhagic stroke was observed (RR 0.65, 95% CrI 0.32–1.25), though substantial heterogeneity and low BF preclude strong conclusions. In contrast to non-procedural bleeding, no significant difference in major bleeding (procedural plus non-procedural major bleeding) was observed (RR 0.95, 95% CrI 0.72–1.22) with P(RR < 1) of 66.2% and BF_01_ of 7.01. Similarly, no difference in CV or unexplained death was detected (RR 0.92, 95% CrI 0.62–1.28; P(RR < 1) = 0.723). Complete study level data and results are presented in Supplementary Supplementary material online, Appendix Results  *S10*.

**Table 2:** Posterior estimates for secondary outcomes

Outcome	Pooled RR	95% CrI Lower	95% CrI Upper	P(RR < 1)	tau (Mean)	BF01	BF10
Ischaemic stroke	1.409	0.952	1.966	0.045	0.196	0.970	1.031
Haemorrhagic stroke	0.654	0.320	1.248	0.924	0.445	0.942	1.061
Major bleeding	0.954	0.726	1.221	0.662	0.115	7.012	0.143
CV/unexplained death	0.924	0.629	1.289	0.723	0.287	5.530	0.181

The table summarizes pooled posterior probability estimates along with heterogeneity and posterior probability of superiority for secondary outcomes. BF, Bayes factor, CrI, credible interval; P(RR < 1), posterior probability that the risk ratio is less than 1, CV, cardiovascular; RR, risk ratio.

### Subgroup analysis based on DOAC era studies

**Figure 4:**
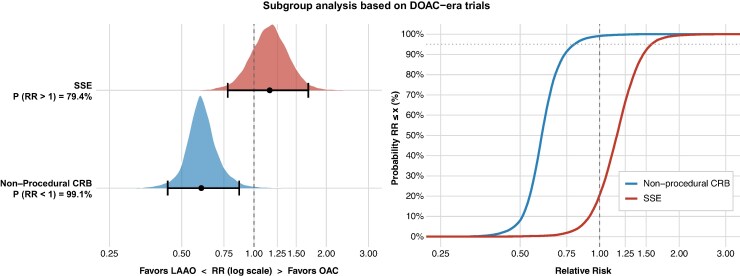
DOAC-era trial subgroup analysis. The left panel shows pooled posterior risk ratio distributions with posterior medians (dots) and 95% credible intervals (horizontal bars); the vertical dashed line marks RR = 1. The right panel shows cumulative posterior probabilities across relative risk thresholds. CRB, clinically relevant bleeding; DOAC, direct oral anticoagulant; OAC, oral anticoagulation; RR, risk ratio; SSE, stroke or systemic embolism.

In a subgroup analysis of the four contemporary trials (PRAGUE-17, OPTION, CLOSURE-AF, and CHAMPION-AF) the pooled RR for stroke or systemic embolism was 1.16 (95% CrI 0.77 to 1.68), and a posterior probability favouring OAC P(RR > 1) of 79.4% (*Figure 4*). The pooled effect for non-procedural CRB was RR 0.60 (0.44 to 0.87) with P(RR < 1) of 99.1%, essentially unchanged from the estimate of the primary analysis.

### Subgroup analysis based on newer generation device studies

**Figure 5:**
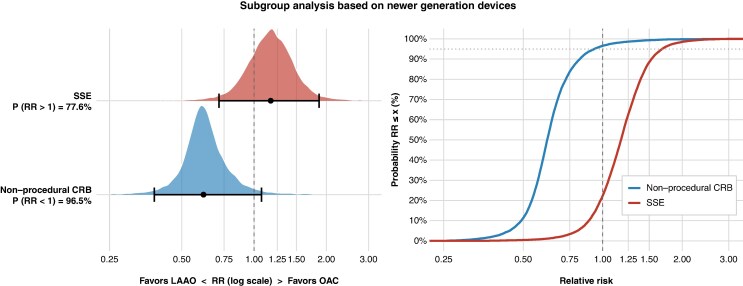
Newer-generation device subgroup analysis. The left panel shows pooled posterior RR distributions with posterior medians (dots) and 95% credible intervals (horizontal bars); the vertical dashed line marks RR = 1. The right panel shows cumulative posterior probabilities across relative risk thresholds. Bleeding favoured device therapy, whereas thromboembolic efficacy remained uncertain. CRB, clinically relevant bleeding; LAAO, left atrial appendage occlusion; RR, risk ratio; SSE, stroke or systemic embolism.

Analysis of trials evaluating the newer generation devices resulted in a pooled RR for SSE of 1.17 (0.71 to 1.86) with P(RR > 1) of 77.6%, and the pooled RR for non-procedural CRB of 0.61 (0.38 to 1.07) with P(RR < 1) of 96.5% (*Figure 5*).

## Discussion

### Summary of principal findings

In this Bayesian meta-analysis of six randomized controlled trials, LAAO was not associated with a reduction in stroke or systemic embolism compared with standard medical therapy. Conversely, LAAO was associated with a definitive reduction in non-procedural CRB. The clinical benefit composite showed a non-significant trend favouring LAAO but was limited by substantial between-study heterogeneity.

### Stroke or systemic embolism

The posterior probability that LAAO reduces SSE relative to OAC was 25.4%, and the Bayes factor (BF_01_) of 5.51 provides moderate strength of evidence in favour of the null. This result is consistent with prior frequentist meta-analyses that reported non-significant differences in SSE between LAAO and OAC.^18,25^ However, the pooled estimates of these analyses warrant careful interpretation. Although the 95% CrI includes the null, its upper bound does not exclude the possibility of an increase in SSE with LAAO, and the Bayesian framework allows us to quantify this directly: the posterior probability that LAAO increases SSE by 25% or more (RR > 1.25) was 19.2%, a non-trivial residual concern. Conversely, the posterior probability of non-inferiority at RR of 1.25 was 80.8% and the probability of equivalence was 78.3%, indicating that superiority of LAAO over OAC for SSE is unlikely with these margins. These posterior probabilities can be contextualized against the Bayesian non-inferiority thresholds used in the landmark trial, PROTECT AF. This trial used a pre-specified threshold of 97.5% for non-inferiority. With the currently accepted margin of HR < 1.4, the posterior probability of non-inferiority of pooled SSE in our analysis was 89%.

Mechanistically, although LAAO addresses thrombus originating from the left atrial appendage it does not mitigate thromboembolic risk from non-LAA sources such as the left atrial body, aortic arch atheroma, or carotid disease.^26,27^ Device-related thrombus, paradoxically increasing short-term embolic risk, might explain some of the attenuation of the net benefit in stroke prevention.^28,29^

### Non-procedural CRB

The reduction in clinically relevant non-procedural bleeding represents one of the most robust and consistent findings in the LAAO evidence base. The posterior probability of P(RR < 1) = 0.999 and a Bayes factor of 30.72 constitute strong evidence by established Bayesian classification thresholds and remain stable across all prior sensitivity analyses. The mechanism underlying this finding is straightforward: LAAO enables discontinuation or substantial reduction of chronic long term oral anticoagulation thereby conferring a durable reduction in haemorrhagic risk beyond the endothelialization phase.^29^ The magnitude of this reduction is clinically meaningful, particularly in patients with elevated bleeding risk (HAS-BLED ≥ 3), in whom annual rates of major bleeding on OAC may exceed 3–5% and the absolute benefit of LAAO would be substantially amplified.^30,31^

However, this result must be interpreted alongside the major bleeding analysis, which includes both procedural and non-procedural events. When periprocedural bleeding complications inherent to the LAAO procedure itself are incorporated, the pooled estimate showed no clear difference between groups (RR 0.95, 95% CrI 0.72–1.22). This decrease in the bleeding advantage in the overall composite is an expected consequence of the procedural risk in the device arm. Whether this trade-off favours the procedure requires careful clinical adjudication at an individual patient level.

### Secondary outcomes and clinical benefit composite

The secondary outcome analyses revealed a pattern consistent with the primary findings. LAAO was associated with a numeric increase in ischaemic stroke (RR 1.40, 95% CrI 0.95 to 1.96) and a numeric decrease in haemorrhagic stroke (RR 0.65, 95% CrI 0.32 to 1.25). However, neither result reached conventional thresholds for statistical credibility i.e. BF were inconclusive for both. Previous meta-analyses have reported a statistically significant decrease in CV or unexplained death with LAAO, however, owing to the numerically higher rates of CV mortality in the device arm of CLOSURE AF, combined analysis after inclusion of these trial results did not result in a significant difference for this outcome.

The clinical benefit composite (RR 0.90, 95% CrI 0.51 to 1.50) must be interpreted with caution. Although the point estimate favours LAAO, the between-study heterogeneity was substantial possibly driven by inherent differences in these composite endpoints.

### Strengths and limitations

Several methodological strengths merit acknowledgment. The Bayesian hierarchical framework provides probability statements that are directly clinically interpretable. Posterior probabilities allow formal distinction between absence of evidence and evidence of absence, especially for outcomes where quantifying weight of evidence of either side of a margin is more meaningful than a non-significant *P*-value. A comprehensive prior sensitivity analysis across six specifications demonstrated that the principal conclusions are not artefacts of prior choice.

However, several limitations warrant consideration. The inclusion of the CLOSURE-AF and CHAMPION AF trials was done after the last date of the initial search strategy. Risk stratified outcomes based on CHA_2_DS_2_-VASc score would allow for a deeper subgroup analysis to study the interaction effects of the treatment across strata and identify patient subgroups most likely to derive a net clinical benefit from LAAO. The equivalence and non-inferiority margins applied here were selected based on conventional regulatory precedents and clinically meaningful estimates. A more rigorous approach would pre-specify posterior probability thresholds *a priori*, as advocated in recent methodological guidance for Bayesian trial design.^32,33^

### Conclusions

This Bayesian meta-analysis demonstrates that the current body of evidence is insufficient to establish clinically meaningful non-inferiority of LAAO over OAC for stroke or systemic embolism. Despite a significant reduction in long-term non-procedural bleeding, LAAO did not demonstrate similar reduction in major bleeding, which includes procedural bleeding. Future trials should consider enrolling homogenous risk profiles and use updated pre-specified margins. Further analyses should plan for a risk-stratified approach to analysing outcomes, which would clarify whether treatment effects differ for patients at the intersection of high stroke and bleed risk.

## Supplementary Material

euag163_Supplementary_Data

## Data Availability

The data underlying this article are available in the article and in its online Supplementary material.

## References

[1] Tan S, Zhou J, Veang T, Lin Q, Liu Q. Global, regional, and national burden of atrial fibrillation and atrial flutter from 1990 to 2021: sex differences and global burden projections to 2046-a systematic analysis of the global burden of disease study 2021. Europace 2025;27:euaf027. 39947238 10.1093/europace/euaf027 PMC11879048

[2] Cheng S, He J, Han Y, Han S, Li P, Liao H et al Global burden of atrial fibrillation/atrial flutter and its attributable risk factors from 1990 to 2021. Europace 2024;26:euae195. 38984719 10.1093/europace/euae195 PMC11287210

[3] Joglar JA, Chung MK, Armbruster AL, Benjamin EJ, Chyou JY, Cronin EM et al 2023 ACC/AHA/ACCP/HRS guideline for the diagnosis and management of atrial fibrillation: a report of the American College of Cardiology/American Heart Association joint committee on clinical practice guidelines. Circulation 2024;149:e1–156. 38033089 10.1161/CIR.0000000000001193 PMC11095842

[4] Ruff CT, Giugliano RP, Braunwald E, Hoffman EB, Deenadayalu N, Ezekowitz MD et al Comparison of the efficacy and safety of new oral anticoagulants with warfarin in patients with atrial fibrillation: a meta-analysis of randomised trials. The Lancet 2014;383:955–62. 10.1016/S0140-6736(13)62343-0 24315724

[5] Feldeisen T, Alexandris-Souphis C, Haymart B, Kong X, Kline-Rogers E, Handoo F et al Anticoagulation changes following Major and clinically relevant nonmajor bleeding events in non-valvular atrial fibrillation patients. J Pharm Pract 2023;36:542–7. 34962835 10.1177/08971900211064189

[6] Reddy VY, Sievert H, Halperin J, Doshi SK, Buchbinder M, Neuzil P et al Percutaneous left atrial appendage closure vs warfarin for atrial fibrillation: a randomized clinical trial. JAMA 2014;312:1988. 25399274 10.1001/jama.2014.15192

[7] Blackshear JL, Johnson WD, Odell JA, Baker VS, Howard M, Pearce L et al Thoracoscopic extracardiac obliteration of the left atrial appendage for stroke risk reduction in atrial fibrillation. JACC 2003;42:1249–52. 14522490 10.1016/s0735-1097(03)00953-7

[8] Odell JA, Blackshear JL, Davies E, Byrne WJ, Kollmorgen CF, Edwards WD et al Thoracoscopic obliteration of the left atrial appendage: potential for stroke reduction? Ann Thorac Surg 1996;61:565–9. 8572768 10.1016/0003-4975(95)00885-3

[9] Van Gelder IC, Rienstra M, Bunting KV, Casado-Arroyo R, Caso V, Crijns HJGM et al 2024 ESC guidelines for the management of atrial fibrillation developed in collaboration with the European association for cardio-thoracic surgery (EACTS): developed by the task force for the management of atrial fibrillation of the European Society of Cardiology (ESC), with the special contribution of the European heart rhythm association (EHRA) of the ESC. Endorsed by the European stroke organisation (ESO). Eur Heart J 2024;45:3314–414. 39210723 10.1093/eurheartj/ehae176

[10] Potpara T, Grygier M, Haeusler KG, Nielsen-Kudsk JE, Berti S, Genovesi S et al Practical guide on left atrial appendage closure for the non-implanting physician. An international consensus paper. Europace 2024;26:euae035. 38291925 10.1093/europace/euae035 PMC11009149

[11] Lakkireddy D, Thaler D, Ellis CR, Swarup V, Sondergaard L, Carroll J et al Amplatzer amulet left atrial appendage occluder versus watchman device for stroke prophylaxis (amulet IDE): a randomized, controlled trial. Circulation 2021;144:1543–52. 34459659 10.1161/CIRCULATIONAHA.121.057063 PMC8570346

[12] Premarket Approval (PMA). Accessed April 7, 2026. https://www.accessdata.fda.gov/scripts/cdrh/cfdocs/cfpma/pma.cfm?id=P130013

[13] Reddy VY, Doshi SK, Kar S, Gibson DN, Price MJ, Huber K et al 5-Year outcomes after left atrial appendage closure. JACC 2017;70:2964–75. 29103847 10.1016/j.jacc.2017.10.021

[14] Osmancik P, Herman D, Neuzil P, Hala P, Taborsky M, Kala P et al 4-Year outcomes after left atrial appendage closure versus nonwarfarin oral anticoagulation for atrial fibrillation. JACC 2022;79:1–14. 34748929 10.1016/j.jacc.2021.10.023

[15] Wazni OM, Saliba WI, Nair DG, Marijon E, Schmidt B, Hounshell T et al Left atrial appendage closure after ablation for atrial fibrillation. N Engl J Med 2025;392:1277–87. 39555822 10.1056/NEJMoa2408308

[16] Doshi SK, Kar S, Nair DG, Waggoner T, Agarwal H, Moussavian M et al Left atrial appendage closure or anticoagulation for atrial fibrillation. N Engl J Med 2026;394:2083–94. 41910347 10.1056/NEJMoa2517213

[17] Landmesser U, Skurk C, Kirchhof P, Lewalter T, Hartung J, Rroku A et al Left atrial appendage closure or medical therapy in atrial fibrillation. N Engl J Med 2026;394:1270–80. 41849741 10.1056/NEJMoa2513310

[18] Kaisaier W, Xu Z, Guo L, Dong Y, Chen Y, Lip GYH et al Left atrial appendage closure vs oral anticoagulation for stroke prevention in atrial fibrillation: long-term outcomes from 4 randomized trials. Heart Rhythm 2025;22:e1086–96. 40754231 10.1016/j.hrthm.2025.07.051

[19] Fernandes JM, Pinheiro RPS, Serpa F, De Andrade NM, Pereira V, Sbardelotto ÂEE et al Left atrial appendage occlusion devices vs direct oral anticoagulants for atrial fibrillation: an updated systematic review and meta-analysis. Curr Probl Cardiol 2025;50:102880. 39395644 10.1016/j.cpcardiol.2024.102880

[20] Franchin L, Piroli F, Demola P, Mantovani F, Iannaccone M, Manfredi R et al Efficacy and safety of left atrial appendage closure compared with oral anticoagulation in atrial fibrillation: a meta-analysis of randomized controlled trials and propensity-matched studies. Front Cardiovasc Med 2023;10:1212161. 37829693 10.3389/fcvm.2023.1212161 PMC10565038

[21] C Iyer S, Yap QV, Soong J, Cove M. Bayesian meta-analysis in the 21st century: fad or future of evidence synthesis? Ann Acad Med Singap 2025;54:437–41. 40799095 10.47102/annals-acadmedsg.2025104

[22] Reis DJ, Kaizer AM, Kinney AR, Bahraini NH, Holliday R, Forster JE et al A practical guide to random-effects Bayesian meta-analyses with application to the psychological trauma and suicide literature. Psychol Trauma Theory Res Pract Policy 2023;15:121–30. 10.1037/tra0001316 PMC10021079 35862085

[23] Berkhout SW, Haaf JM, Gronau QF, Heck DW, Wagenmakers EJ. A tutorial on Bayesian model-averaged meta-analysis in JASP. Behav Res Methods 2023;56:1260–82. 37099263 10.3758/s13428-023-02093-6 PMC10991068

[24] Chandler J, McKenzie J, Boutron I, Welch V Cochrane Methods 2016. UK: Wiley; 2016.

[25] Oliva A, Ioppolo AM, Chiarito M, Cremonesi A, Azzano A, Miccichè E et al Left atrial appendage closure compared with oral anticoagulants for patients with atrial fibrillation: a systematic review and network meta-analysis. J Am Heart Assoc 2024;13:e034815. 39119987 10.1161/JAHA.124.034815 PMC11963956

[26] Kamel H, Okin PM, Elkind MSV, Iadecola C. Atrial fibrillation and mechanisms of stroke: time for a new model. Stroke 2016;47:895–900. 26786114 10.1161/STROKEAHA.115.012004 PMC4766055

[27] Maarse M, Boersma LVA, Swaans MJ. Thrombi outside the left atrial appendage: “small potatoes”? EuroIntervention 2019;15:e216–8. 31186222 10.4244/EIJV15I3A39

[28] Alkhouli M, Busu T, Shah K, Osman M, Alqahtani F, Raybuck B. Incidence and clinical impact of device-related thrombus following percutaneous left atrial appendage occlusion. JACC Clin Electrophysiol 2018;4:1629–37. 30573129 10.1016/j.jacep.2018.09.007

[29] Dukkipati SR, Kar S, Holmes DR, Doshi SK, Swarup V, Gibson DN et al Device-related thrombus after left atrial appendage closure: incidence, predictors, and outcomes. Circulation 2018;138:874–85. 29752398 10.1161/CIRCULATIONAHA.118.035090

[30] Pisters R, Lane DA, Nieuwlaat R, De Vos CB, Crijns HJGM, Lip GYH. A novel user-friendly score (HAS-BLED) to assess 1-year risk of Major bleeding in patients with atrial fibrillation. Chest 2010;138:1093–100. 20299623 10.1378/chest.10-0134

[31] Lip GYH, Frison L, Halperin JL, Lane DA. Comparative validation of a novel risk score for predicting bleeding risk in anticoagulated patients with atrial fibrillation. J Am Coll Cardiol 2011;57:173–80. 21111555 10.1016/j.jacc.2010.09.024

[32] Use of Bayesian Methodology in Clinical Trials of Drug and Biological Products: Draft Guidance for Industry. Published online January 9, 2026. https://www.regulations.gov/document/FDA-2025-D-3217

[33] Ades T . Bayesian approaches to clinical trials and health-care evaluation by D.J. Spiegelhalter, K.R. Abrams and J.P. Miles. Biometrics 2006;62:306–7.

