# Supplementary material for: Comparative evaluation of left atrial appendage occlusion and oral anticoagulation: a Bayesian meta-analysis of randomized controlled trials
Source: Europace. 2026 Jul 23;28(7):euag163. doi: 10.1093/europace/euag163 (PMC13391208; doi:10.1093/europace/euag163)
Supplement: euag163_Supplementary_Data [file euag163_supplementary_data.docx]

**SUPPLEMENTARY APPENDIX**

Comparative Evaluation of Left Atrial Appendage Occlusion And Oral Anticoagulation:

A Bayesian Meta-Analysis of Randomized Controlled Trials

Krishna Saketh Athmakuri MBBS¹; Shreyas Nandyal MBBS²; Hrushikesh Reddy Pamreddy MBBS³; Saketh Vinjamuri MBBS^4^; Shanmukh Sai Pavan Lingamsetty MBBS^5^ ; Anushka Vishwas Mahajan MBBS^1^ ; Muqqaddas Hussain MBBS^1^ ; Hari P. Chaliki, MD^1^ ; Komandoor Srivathsan MD¹.

**Affiliations:**

1.Department of Cardiovascular Medicine, Mayo Clinic Arizona, USA, 85054.

2.Department of Internal Medicine, John H Stroger Jr. Hospital of Cook County, IL, USA.

3.Department of Internal Medicine, Saint Vincent Hospital, Worcester MA, USA.

4.Department of Internal Medicine, Cleveland Clinic Fairview Hospital, Cleveland, OH, USA.

5. Division of Medicine, Beth Israel Deaconess Medical Center, Boston, MA.

*Table of Contents*

[Supplementary Appendix Methods S1 PRISMA Checklist 3](#_Toc230872765)

[Supplementary Appendix Methods S2. PRISMA Abstract Checklist 6](#_Toc230872766)

[Supplementary Appendix Methods S3 Search Strategy 7](#_Toc230872767)

[Supplementary Material Methods S4 Exclusion with reasons 10](#_Toc230872768)

[Supplementary Appendix Methods S5 Outcome Definitions 13](#_Toc230872769)

[Supplementary Appendix Methods S6 Risk of Bias Assessment 14](#_Toc230872770)

[Stroke / Systemic Embolism 14](#_Toc230872771)

[Clinically Relevant Bleeding 15](#_Toc230872772)

[Supplementary Appendix Methods S7 Publication Bias 16](#_Toc230872773)

[Funnel Plots 16](#_Toc230872774)

[Supplementary Appendix Methods S8 Statistical analysis 18](#_Toc230872775)

[Prior specifications 18](#_Toc230872776)

[Convergence Diagnostics 20](#_Toc230872777)

[Trace Plots 21](#_Toc230872778)

[Posterior Predictive Check Plots 23](#_Toc230872779)

[Supplementary Appendix Results S9 Sensitivity analysis 25](#_Toc230872780)

[Supplementary Appendix Results S10 Additional results 26](#_Toc230872781)

[Study level data 26](#_Toc230872782)

[Study Level Plots 29](#_Toc230872783)

[Supplementary Appendix Results S11 References 31](#_Toc230872784)

Supplementary Appendix Methods S1 PRISMA Checklist

| **Topic** | | **No.** | | **Item** | | | | **Location where item is reported** |
| --- | --- | --- | --- | --- | --- | --- | --- | --- |
| **TITLE** | | | | | | | | |
| **Title** | | 1 | | Identify the report as a systematic review. | | | | pg. 1 in the MS |
| **ABSTRACT** | | | | | | | | |
| **Abstract** | | 2 | | See the PRISMA 2020 for Abstracts checklist | | | |  |
| **INTRODUCTION** | | | | | | | | |
| **Rationale** | | 3 | | Describe the rationale for the review in the context of existing knowledge. | | | | pg. 7 in the MS |
| **Objectives** | | 4 | | Provide an explicit statement of the objective(s) or question(s) the review addresses. | | | | pg. 8 in the MS |
| **METHODS** | | | | | | | | |
| **Eligibility criteria** | | 5 | | Specify the inclusion and exclusion criteria for the review and how studies were grouped for the syntheses. | | | | pg. 9 in the MS |
| **Information sources** | | 6 | | Specify all databases, registers, websites, organisations, reference lists and other sources searched or consulted to identify studies. Specify the date when each source was last searched or consulted. | | | | pg. 10 in the MS |
| **Search strategy** | | 7 | | Present the full search strategies for all databases, registers and websites, including any filters and limits used. | | | | pg. 7 in the suppl. |
|  | |  | | Specify the methods used to decide whether a study met the inclusion criteria of the review, including how many reviewers | | | |  |
| **Selection process** | | 8 | | screened each record and each report retrieved, whether they worked independently, and if applicable, details of automation | | | | pg. 10 in the MS |
|  | |  | | tools used in the process. | | | |  |
|  | |  | | Specify the methods used to collect data from reports, including how many reviewers collected data from each report, whether | | | |  |
| **Data collection process** | | 9 | | they worked independently, any processes for obtaining or confirming data from study investigators, and if applicable, details | | | | pg. 10 in the MS |
|  | |  | | of automation tools used in the process. | | | |  |
| **Data items** | | 10a | | List and define all outcomes for which data were sought. Specify whether all results that were compatible with each outcome domain in each study were sought (e.g. for all measures, time points, analyses), and if not, the methods used to decide which  results to collect. | | | | pg. 10 in the MS; pg. 8-9 in the suppl. |
|  | | 10b | | List and define all other variables for which data were sought (e.g. participant and intervention characteristics, funding sources). Describe any assumptions made about any missing or unclear information. | | | | pg. 11 in the MS |
| **Study risk of bias assessment** | | 11 | | Specify the methods used to assess risk of bias in the included studies, including details of the tool(s) used, how many reviewers assessed each study and whether they worked independently, and if applicable, details of automation tools used in the process. | | | | pg. 11 in the MS |
| **Effect measures** | | 12 | | Specify for each outcome the effect measure(s) (e.g. risk ratio, mean difference) used in the synthesis or presentation of results. | | | | pg. 12 in the MS |
| **Topic** | | No. | | Item | | | | Location where item is reported |
| **Synthesis methods** | | 13a | | Describe the processes used to decide which studies were eligible for each synthesis (e.g. tabulating the study intervention characteristics and comparing against the planned groups for each synthesis (item 5)). | | | | pg..10-11 in the MS; pg. 10 in the suppl. |
|  | | 13b | | Describe any methods required to prepare the data for presentation or synthesis, such as handling of missing summary statistics, or data conversions. | | | | pg..11-12 in the MS; pg. 10 in the suppl. |
|  | | 13c | | Describe any methods used to tabulate or visually display results of individual studies and syntheses. | | | | pg..10-11 in the MS; pg. 10 in the suppl. |
|  | | 13d | | Describe any methods used to synthesize results and provide a rationale for the choice(s). If meta-analysis was performed, describe the model(s), method(s) to identify the presence and extent of statistical heterogeneity, and software package(s) used. | | | | pg..14 in the MS; pg. 10 in the suppl. |
|  | | 13e | | Describe any methods used to explore possible causes of heterogeneity among study results (e.g. subgroup analysis, meta- regression). | | | | pg. 13 in the MS; |
|  | | 13f | | Describe any sensitivity analyses conducted to assess robustness of the synthesized results. | | | | pg. 26 in the Suppl; |
| **Reporting bias assessment** | | 14 | | Describe any methods used to assess risk of bias due to missing results in a synthesis (arising from reporting biases). | | | | NA; |
| **Certainty assessment** | | 15 | | Describe any methods used to assess certainty (or confidence) in the body of evidence for an outcome. | | | | NA |
| **RESULTS** | | | | | | | | |
| **Study selection** | | | 16a | | Describe the results of the search and selection process, from the number of records identified in the search to the number of studies included in the review, ideally using a flow diagram. | | fig. 1 | |
|  | | | 16b | | Cite studies that might appear to meet the inclusion criteria, but which were excluded, and explain why they were excluded. | | pg.10 Suppl | |
| **Study characteristics** | | | 17 | | Cite each included study and present its characteristics. | | Pg. 14 in the MS; table 1 | |
| **Risk of bias in studies** | | | 18 | | Present assessments of risk of bias for each included study. | | pg. 14 in the suppl. | |
| **Results of individual studies** | | | 19 | | For all outcomes, present, for each study: (a) summary statistics for each group (where appropriate) and (b) an effect estimates and its precision (e.g. confidence/credible interval), ideally using structured tables or plots. | | Table 2-fig.2 in the MS; fig. S3-S6; pg. 18-21 in the suppl. | |
| **Results of syntheses** | | | 20a | | For each synthesis, briefly summarise the characteristics and risk of bias among contributing studies. | | pg. 13-14 in the suppl. | |
|  | | | 20b | | Present results of all statistical syntheses conducted. If meta-analysis was done, present for each the summary estimate and its precision (e.g. confidence/credible interval) and measures of statistical heterogeneity. If comparing groups, describe the Immediateion of the effect. | | Table 2- fig. 2in the MS; pg. 11, 13 in the suppl. | |
|  | | | 20c | | Present results of all investigations of possible causes of heterogeneity among study results. | | Table 2; table 3; pg. 7-8 in the MS., pg. 20-25 in the suppl. | |
|  | | | 20d | | Present results of all sensitivity analyses conducted to assess the robustness of the synthesized results. | | pg. 7-8 in the MS; pg. 18- 25 in the suppl. | |
| **Reporting biases** | | | 21 | | Present assessments of risk of bias due to missing results (arising from reporting biases) for each synthesis assessed. | | pg. 14 in the suppl. | |
| **Certainty of evidence** | | | 22 | | Present assessments of certainty (or confidence) in the body of evidence for each outcome assessed. | | pg. 26 in the suppl. | |
|  | | |  | |  | |  | |
| **Topic** | **No.** | **Item** | | | | **Location where item is reported** | | |
| **DISCUSSION** | | | | | | | | |
| **Discussion** | 23a | Provide a general interpretation of the results in the context of other evidence. | | | | pg. 18 in the MS | | |
|  | 23b | Discuss any limitations of the evidence included in the review. | | | | pg. 22 in the MS | | |
|  | 23c | Discuss any limitations of the review processes used. | | | | pg. 22 in the MS | | |
|  | 23d | Discuss implications of the results for practice, policy, and future research. | | | | pg. 23 in the MS | | |
| **OTHER INFORMATION** | | | | | | | | |
| **Registration and protocol** | 24a | Provide registration information for the review, including register name and registration number, or state that the review was not registered. | | | | PROSPERO; ID [CRD420251266412]) | | |
|  | 24b | Indicate where the review protocol can be accessed, or state that a protocol was not prepared. | | | | [YES](https://www.crd.york.ac.uk/prospero/display_record.php?ID=CRD42024534705) | | |
|  | 24c | Describe and explain any amendments to information provided at registration or in the protocol. | | | | None. | | |
| **Support** | 25 | Describe sources of financial or non-financial support for the review, and the role of the funders or sponsors in the review. | | | | None | | |
| **Competing interests** | 26 | Declare any competing interests of review authors. | | | | pg. 2 in the MS; | | |
| **Availability of data, code and other**  **materials** | 27 | Report which of the following are publicly available and where they can be found: template data collection forms; data extracted from included studies; data used for all analyses; analytic code; any other materials used in the review. | | | | Will be produced on request | | |

Abbreviations: MS, manuscript; suppl., supplement

Supplementary Appendix Methods S2. PRISMA Abstract Checklist

| **Topic** | **No.** | **Item** | **Reported?** |
| --- | --- | --- | --- |
| **TITLE** | | | |
| **Title** | 1 | Identify the report as a systematic review. | Yes |
| **BACKGROUND** | | | |
| **Objectives** | 2 | Provide an explicit statement of the main objective(s) or question(s) the review addresses. | Yes |
| **METHODS** | | | |
| **Eligibility criteria** | 3 | Specify the inclusion and exclusion criteria for the review. | Yes |
| **Information sources** | 4 | Specify the information sources (e.g. databases, registers) used to identify studies and the date when each was last searched. | Yes |
| **Risk of bias** | 5 | Specify the methods used to assess risk of bias in the included studies. | No |
| **Synthesis of results** | 6 | Specify the methods used to present and synthesize results. | Yes |
| **RESULTS** | | | |
| **Included studies** | 7 | Give the total number of included studies and participants and summarise relevant characteristics of studies. | Yes |
| **Synthesis of results** | 8 | Present results for main outcomes, preferably indicating the number of included studies and participants for each. If meta-analysis was done, report the summary estimate and confidence/credible interval. If comparing groups, indicate the Immediateion of the effect (i.e. which group is favoured). | Yes |
| **DISCUSSION** | | | |
| **Limitations of evidence** | 9 | Provide a brief summary of the limitations of the evidence included in the review (e.g. study risk of bias, inconsistency and imprecision). | No |
| **Interpretation** | 10 | Provide a general interpretation of the results and important implications. | Yes |
| **OTHER** | | | |
| **Funding** | 11 | Specify the primary source of funding for the review. | No |
| **Registration** | 12 | Provide the register name and registration number. | No |

Supplementary Appendix Methods S3 Search Strategy

**12.23.25 PubMed (959)** (((((("Atrial Fibrillation"[Mesh]) OR ("atrial fibrillation" OR afib OR af)) OR ("non-valvular atrial fibrillation")) OR (nvaf)) AND ((((("Left Atrial Appendage Closure"[Mesh]) OR ("Septal Occluder Device"[Mesh])) OR ("left atrial appendage closure")) OR ("left atrial appendage occlusion")) OR (laao OR laac OR watchman OR "amplatzer amulet" OR lariat))) AND (("Anticoagulants"[Mesh] OR "Anticoagulants" [Pharmacological Action]) OR (oac OR vka OR noac OR doac)) AND (english[Filter])) OR (((((("Atrial Fibrillation"[Mesh]) OR ("atrial fibrillation" OR afib OR af)) OR ("non-valvular atrial fibrillation")) OR (nvaf)) AND ((((("Left Atrial Appendage Closure"[Mesh]) OR ("Septal Occluder Device"[Mesh])) OR ("left atrial appendage closure")) OR ("left atrial appendage occlusion")) OR (laao OR laac OR watchman OR "amplatzer amulet" OR lariat))) AND (("Anticoagulants"[Mesh] OR "Anticoagulants" [Pharmacological Action]) OR (oac OR vka OR noac OR doac)) AND ((randomizedcontrolledtrial[Filter]) AND (english[Filter])))

**12.24.25 Embase <1974 to 2025 December 22>**
#, Query, Results from 24 Dec 2025

1, exp atrial fibrillation/, 265,088

2, (non-valvular adj1 atrial adj1 fibrillation).mp. [mp=title, abstract, heading word, drug trade name, original title, device manufacturer, drug manufacturer, device trade name, keyword heading word, floating subheading word, candidate term word], 6,156

3, (afib or nvaf).mp. [mp=title, abstract, heading word, drug trade name, original title, device manufacturer, drug manufacturer, device trade name, keyword heading word, floating subheading word, candidate term word], 6,456

4, 1 or 2 or 3, 266,247

5, (left adj1 atrial adj1 appendage adj1 occlusion).mp. [mp=title, abstract, heading word, drug trade name, original title, device manufacturer, drug manufacturer, device trade name, keyword heading word, floating subheading word, candidate term word], 3,233

6, (left adj1 atrial adj1 appendage adj1 closure).mp. [mp=title, abstract, heading word, drug trade name, original title, device manufacturer, drug manufacturer, device trade name, keyword heading word, floating subheading word, candidate term word], 7,311

7, exp left atrial appendage closure device/ or left atrial appendage closure/, 6,585

8, watchman.mp., 3,324

9, (laao or laac).mp. [mp=title, abstract, heading word, drug trade name, original title, device manufacturer, drug manufacturer, device trade name, keyword heading word, floating subheading word, candidate term word], 3,311

10, (amplatzer adj1 amulet).mp. [mp=title, abstract, heading word, drug trade name, original title, device manufacturer, drug manufacturer, device trade name, keyword heading word, floating subheading word, candidate term word], 514

11, (lariat adj1 device).mp. [mp=title, abstract, heading word, drug trade name, original title, device manufacturer, drug manufacturer, device trade name, keyword heading word, floating subheading word, candidate term word], 122

12, 5 or 6 or 7 or 8 or 9 or 10 or 11, 8,829

13, 4 and 12, 5,731

14, exp anticoagulant agent/ or anticoagulant.mp., 934,633

15, (warfarin or dabigatran or rivaroxaban or Apixaban or edoxaban or OAC or vka or noac or doac).mp. [mp=title, abstract, heading word, drug trade name, original title, device manufacturer, drug manufacturer, device trade name, keyword heading word, floating subheading word, candidate term word], 174,029

16, 14 or 15, 945,549

17, 13 and 16, 3,556

18, randomized control* trial*.mp. or exp randomized controlled trial/, 1,509,083

19, 17 and 18, 532

20, limit 19 to english language, 523

**12.24.25 Scopus (1134)** ( ALL ( rct OR "randomized controlled trial*" ) ) AND ( ( ( TITLE-ABS-KEY ( "atrial fibrillation" OR afib OR "non-valvular atrial fibrillation" OR nvaf ) ) AND ( ( TITLE-ABS-KEY ( "left atrial appendage" OR "septal occulder device" OR "left atrial appendage closure" OR "left atrial apendage occlusion" ) OR TITLE-ABS-KEY ( laao OR laac OR watchman OR "amplatzer amulet" OR "lariat device" ) ) ) ) AND ( TITLE-ABS-KEY ( anticoagulant OR oac OR vka OR noac OR doac OR warfarin OR dabigatran OR rivaroxaban OR apixaban OR edoxaban ) ) ) AND ( LIMIT-TO ( LANGUAGE , "English" ) )

**12.23.25 Web of Science (77)** "atrial fibrillation" OR afib OR "non-valvular atrial fibrillation" OR nvaf (Topic) AND "left atrial appendage closure" OR "septal occluder device*" OR "left atrial appendage closure" OR "left atrial appendage occlusion" OR laao OR laac OR watchman OR "amplatzer amulet" OR lariat (Topic) AND anticoagulant OR oac OR vka OR noac OR doac OR warfarin OR dabigatran OR rivaroxaban OR apixaban OR edoxaban (All Fields) AND "randomized controlled trial*" OR rct (All Fields) and and English (Languages)

**12.24.25 EBM Reviews - Cochrane Central Register of Controlled Trials <November 2025>**

#, Query, Results from 24 Dec 2025

1, exp atrial fibrillation/, 7,573

2, (non-valvular adj1 atrial adj1 fibrillation).mp. [mp=title, original title, abstract, floating sub-heading word, mesh headings, heading words, keyword], 545

3, (afib or nvaf).mp. [mp=title, original title, abstract, floating sub-heading word, mesh headings, heading words, keyword], 411

4, 1 or 2 or 3, 8,030

5, (left adj1 atrial adj1 appendage adj1 occlusion).mp. [mp=title, original title, abstract, floating sub-heading word, mesh headings, heading words, keyword], 187

6, (left adj1 atrial adj1 appendage adj1 closure).mp. [mp=title, original title, abstract, floating sub-heading word, mesh headings, heading words, keyword], 266

7, exp left atrial appendage closure device/ or left atrial appendage closure/, 9

8, watchman.mp., 151

9, (laao or laac).mp. [mp=title, original title, abstract, floating sub-heading word, mesh headings, heading words, keyword], 213

10, (amplatzer adj1 amulet).mp. [mp=title, original title, abstract, floating sub-heading word, mesh headings, heading words, keyword], 43

11, (lariat adj1 device).mp. [mp=title, original title, abstract, floating sub-heading word, mesh headings, heading words, keyword], 2

12, 5 or 6 or 7 or 8 or 9 or 10 or 11, 429

13, 4 and 12, 223

14, exp anticoagulant agent/ or anticoagulant.mp., 20,419

15, (warfarin or dabigatran or rivaroxaban or Apixaban or edoxaban or OAC or vka or noac or doac).mp. [mp=title, original title, abstract, floating sub-heading word, mesh headings, heading words, keyword], 9,624

16, 14 or 15, 24,624

17, 13 and 16, 132

18, randomized control* trial*.mp. or exp randomized controlled trial/, 739,118

19, 17 and 18, 71

20, limit 19 to english language, 66

**12.24.25 ClinicalTrials.gov (23)** Non-valvular atrial fibrillation \(nvaf\) | Anticoagulants

| DATABASE | RESULTS | DUPLICATES | REMAINING |
| --- | --- | --- | --- |
| PubMed | 959 | 10 | 949 |
| Embase | 523 | 179 | 344 |
| Scopus | 1134 | 587 | 547 |
| Web of Science | 77 | 67 | 10 |
| Cochrane | 66 | 62 | 4 |
| ClinicalTrials.gov | 23 | 8 | 15 |
| **TOTAL** | 2782 | 913 | 1869 |
|  |  |  |  |

Supplementary Material Methods S4 Exclusion with reasons

| **Authors** | **Year** | **Journal** | **Title** | **TiAb Screening** | **FT Screening** | **Reason for Exclusion** |
| --- | --- | --- | --- | --- | --- | --- |
| Boersma et al. | 2017 | Heart Rhythm | Efficacy and safety of left atrial appendage closure with WATCHMAN in patients with or without contraindication to oral anticoagulation: 1-Year follow-up outcome data of the EWOLUTION trial | 1 | 0 | Not an RCT; single-arm prospective registry |
| Branny et al. | 2023 | J Cardiovasc Electrophysiol | Nonprocedural bleeding after left atrial appendage closure versus direct oral anticoagulants: A subanalysis of the randomized PRAGUE-17 trial | 1 | 0 | Sub-study of included trial (PRAGUE-17) |
| Brouwer et al. | 2019 | JAHA | Net Clinical Benefit of Left Atrial Appendage Closure Versus Warfarin in Patients With Atrial Fibrillation: A Pooled Analysis of the Randomized PROTECT-AF and PREVAIL Studies | 1 | 0 | Pooled post hoc analysis of included trials (PROTECT-AF and PREVAIL) |
| Dukkipati et al. | 2022 | JACC | Impact of Peridevice Leak on 5-Year Outcomes After Left Atrial Appendage Closure | 1 | 0 | Post hoc analysis within device arm only; not LAAO vs OAC comparison |
| Dutta et al. | 2023 | J Interv Card Electrophysiol | High rates of oral anticoagulation in atrial fibrillation patients observed in a large multi-specialty health system in the Northeast | 1 | 0 | Not an RCT; cross-sectional observational study; LAAO not studied as intervention |
| Elsheikh et al. | 2025 | Cerebrovasc Dis | Left Atrial Appendage Occlusion versus Direct Oral Anticoagulants in the Prevention of Ischaemic Stroke in Patients with Atrial Fibrillation | 1 | 0 | Not an RCT; retrospective observational study with propensity score matching |
| Friedman et al. | 2022 | JACC Cardiovasc Interv | Patient-Level Analysis of Watchman Left Atrial Appendage Occlusion in Practice Versus Clinical Trials | 1 | 0 | Not an RCT; propensity-matched trial-vs-registry comparison |
| Fu et al. | 2022 | J Formosan Med Assoc | Safety and efficacy of low-dose non-vitamin K antagonist oral anticoagulants versus warfarin after left atrial appendage closure with the Watchman device | 1 | 0 | Not an RCT; retrospective observational; wrong comparator (post-LAAC anticoagulation regimens) |
| Galea et al. | 2022 | Circulation | Amulet or Watchman Device for Percutaneous Left Atrial Appendage Closure: Primary Results of the SWISS-APERO Randomized Clinical Trial | 1 | 0 | Wrong comparator: device vs device (Amulet vs Watchman), not LAAO vs OAC |
| Gilhofer et al. | 2025 | Swiss Med Wkly | Safety and effectiveness of left atrial appendage occlusion in patients with atrial fibrillation and high bleeding risk: a cardinality-matched comparison with direct oral anticoagulation | 1 | 0 | Not an RCT; observational cardinality-matched cohort study |
| Holmes et al. | 2015 | JACC | Left Atrial Appendage Closure as an Alternative to Warfarin for Stroke Prevention in Atrial Fibrillation: A Patient-Level Meta-Analysis | 1 | 0 | Patient-level meta-analysis of included trials and registries, not an independent RCT |
| Kar et al. | 2021 | Circulation | Primary Outcome Evaluation of a Next-Generation Left Atrial Appendage Closure Device: Results From the PINNACLE FLX Trial | 1 | 0 | Not an RCT; single-arm study with no comparator group |
| Lakkireddy et al. | 2021 | Circulation | Amplatzer Amulet Left Atrial Appendage Occluder Versus Watchman Device for Stroke Prophylaxis (Amulet IDE): A Randomized, Controlled Trial | 1 | 0 | Wrong comparator: device vs device (Amulet vs Watchman), not LAAO vs OAC |
| Lakkireddy et al. | 2025 | JACC | 5-Year Results From the AMPLATZER Amulet Left Atrial Appendage Occluder Randomized Controlled Trial (Amulet IDE) | 1 | 0 | Wrong comparator: device vs device (Amulet vs Watchman), not LAAO vs OAC |
| Melillo et al. | 2023 | Int J Cardiol | Direct oral anticoagulants versus percutaneous left atrial appendage occlusion in atrial fibrillation: 5-year outcomes | 1 | 0 | Not an RCT; observational non-randomized study with propensity score matching |
| Price et al. | 2015 | JACC Cardiovasc Interv | Bleeding Outcomes After Left Atrial Appendage Closure Compared With Long-Term Warfarin: A Pooled, Patient-Level Analysis of the WATCHMAN Randomized Trial Experience | 1 | 0 | Pooled patient-level analysis of included trials (PROTECT-AF and PREVAIL) |
| Price et al. | 2022 | JACC Cardiovasc Interv | Peridevice Leak After Transcatheter Left Atrial Appendage Occlusion: An Analysis of the Amulet IDE Trial | 1 | 0 | Wrong comparator: device vs device; post hoc sub-analysis of Amulet IDE |
| Rodes-Cabau et al. | 2025 | Circulation | Short-Term Anticoagulation Versus Dual Antiplatelet Therapy for Preventing Device Thrombosis Following Left Atrial Appendage Closure: The ANDES Randomized Clinical Trial | 1 | 0 | Wrong comparator: post-LAAC antithrombotic strategy comparison (DOAC vs DAPT), not LAAO vs OAC |
| Sulaiman et al. | 2023 | JACC Clin Electrophysiol | Left Atrial Appendage Occlusion in the Elderly: Insights From PROTECT-AF, PREVAIL, and Continuous Access Registries | 1 | 0 | Pooled sub-group analysis of included trials and non-randomized registries |
| Whang et al. | 2018 | J Atrial Fibrillation | Does Left Atrial Appendage Closure Reduce Mortality? A Vital Status Analysis of the Randomized PROTECT AF and PREVAIL Clinical Trials | 1 | 0 | Retrospective pooled analysis of included trials (PROTECT-AF and PREVAIL) |
| Whitlock et al. | 2021 | NEJM | Left Atrial Appendage Occlusion during Cardiac Surgery to Prevent Stroke (LAAOS III) | 1 | 0 | Wrong intervention: surgical (not percutaneous) LAA occlusion |
| Zhu & Xu | 2021 | Drug Des Devel Ther | The Use of Novel Non-Vitamin K Antagonist Oral Anticoagulants Following Closure of the Left Atrial Appendage: Preliminary Results of Clinical Follow-Up | 1 | 0 | Not an RCT; retrospective observational; wrong comparator (post-LAAC anticoagulation regimens) |

Supplementary Appendix Methods S5 Outcome Definitions

| Outcome | PROTECT-AF | PREVAIL | PRAGUE-17 | OPTION | CHAMPION-AF | CLOSURE-AF |
| --- | --- | --- | --- | --- | --- | --- |
| EFFICACY OUTCOMES- STROKE EVENTS | | | | | | |
| Stroke or systemic embolism | All stroke or systemic embolism | All stroke or systemic embolism | All stroke, systemic embolism | Stroke and systemic embolism( Number of ischemic stroke, hemorrhagic stroke, and systemic embolism in both the groups) | Stroke and systemic embolism counts | Stroke or systemic embolism composite |
| Ischemic stroke | Trial-adjudicated ischemic stroke events | Trial-adjudicated ischemic stroke events | Trial-adjudicated ischemic stroke events | Trial-adjudicated ischemic stroke events | Trial-adjudicated ischemic stroke events | Trial-adjudicated ischemic stroke events |
| Hemorrhagic stroke | Trial-adjudicated hemorrhagic stroke events | Trial-adjudicated hemorrhagic stroke events | Trial-adjudicated hemorrhagic stroke events | Trial-adjudicated hemorrhagic stroke events | Trial-adjudicated hemorrhagic stroke events | Trial-adjudicated hemorrhagic stroke events |
| CV / unexplained death | Cardiovascular or unexplained death | Cardiovascular or unexplained death | Cardiovascular death | Cardiovascular or unexplained death | Cardiovascular or unexplained death | Cardiovascular or unexplained death |
| SAFETY OUTCOMES — BLEEDING | | | | | | |
| Non-procedural clinically relevant bleeding^$^ | Non-procedural major bleeding: intracranial hemorrhage or bleeding requiring transfusion | Non-procedural major bleeding: intracranial hemorrhage or bleeding requiring transfusion | Non-procedural CRB: ISTH major bleeding + clinically relevant non-major bleeding (hospitalization or invasive procedure) | Non-procedure-related CRB: ISTH major + clinically relevant non-major bleeding (medical intervention, hospitalization, or face-to-face evaluation) | Non-procedure-related CRB: ISTH major + clinically relevant non-major bleeding (3-year follow-up) | Non-procedural major bleeding: BARC type 3 or higher |
| Major bleeding^§^ | Intracranial hemorrhage or bleeding requiring transfusion (procedural + non-procedural) | Intracranial hemorrhage or bleeding requiring transfusion (procedural + non-procedural) | Not reported separately. Trial reports only CRB composite (major + non-major combined). Excluded from major bleeding pooling. | ISTH major bleeding: Hb drop ≥2 g/dL, transfusion ≥2 U pRBC, critical-site bleeding, or fatal (procedural + non-procedural) | ISTH major bleeding: procedure-related and non-procedure-related reported separately at 3 years | BARC type 3+  BARC type 3 or higher: overt bleeding with Hb drop ≥3 g/dL, transfusion, surgical intervention, or fatal/intracranial |

Abbreviations: BARC = Bleeding Academic Research Consortium; CRB = clinically relevant bleeding; Hb = hemoglobin; ISTH = International Society on Thrombosis and Haemostasis; pRBC = packed red blood cells; TIA = transient ischemic attack.

$ This outcome is a trial defined composite of non-procedural Major bleeding plus clinically relevant non-major bleeding. This reflects a composite of Major and/or Non-Major Bleeding events.

Note: Non-procedural bleeding excludes procedure-related events to reflect the long-term safety profile of each strategy. For PROTECT-AF and PREVAIL, bleeding definitions predate the ISTH standardized framework. PROTECT, PREVIAL and CLOSURE report Major bleeding events, and not a composite of major and non-major as the other trials

§ Major bleeding events across procedural and non-procedural settings.Trial-defined adjudicated definitions for major bleeding were used. Outcomes reported exclusively as major bleeding were extracted

Supplementary Appendix Methods S6 Risk of Bias Assessment

## Stroke / Systemic Embolism


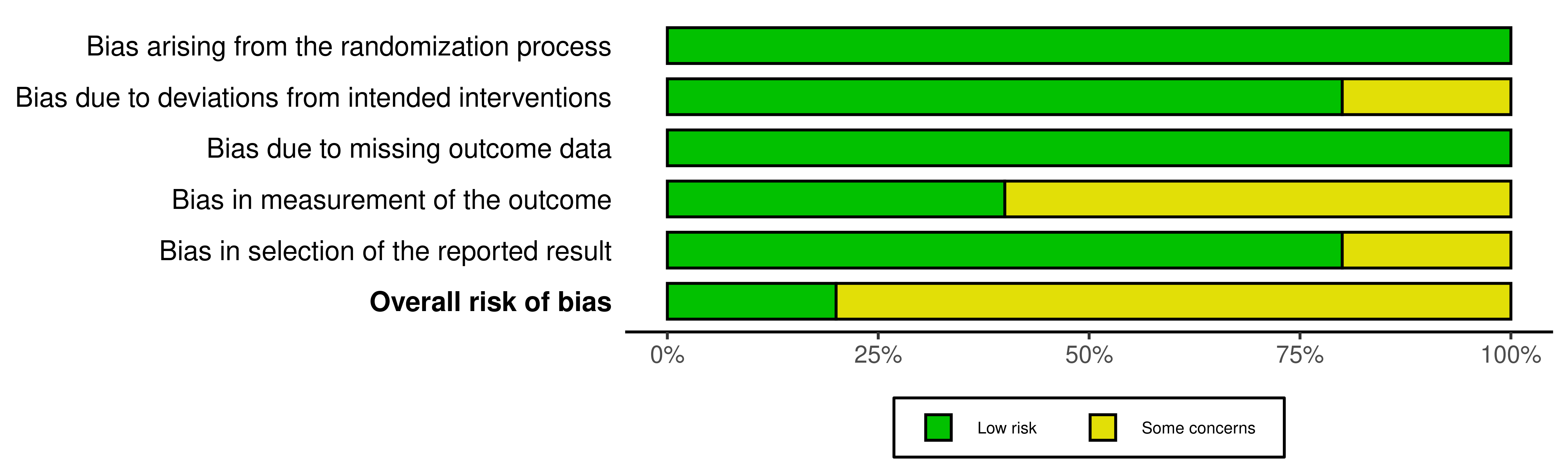


*Figure S1: Summary risk of bias plot for stroke/systemic embolism*


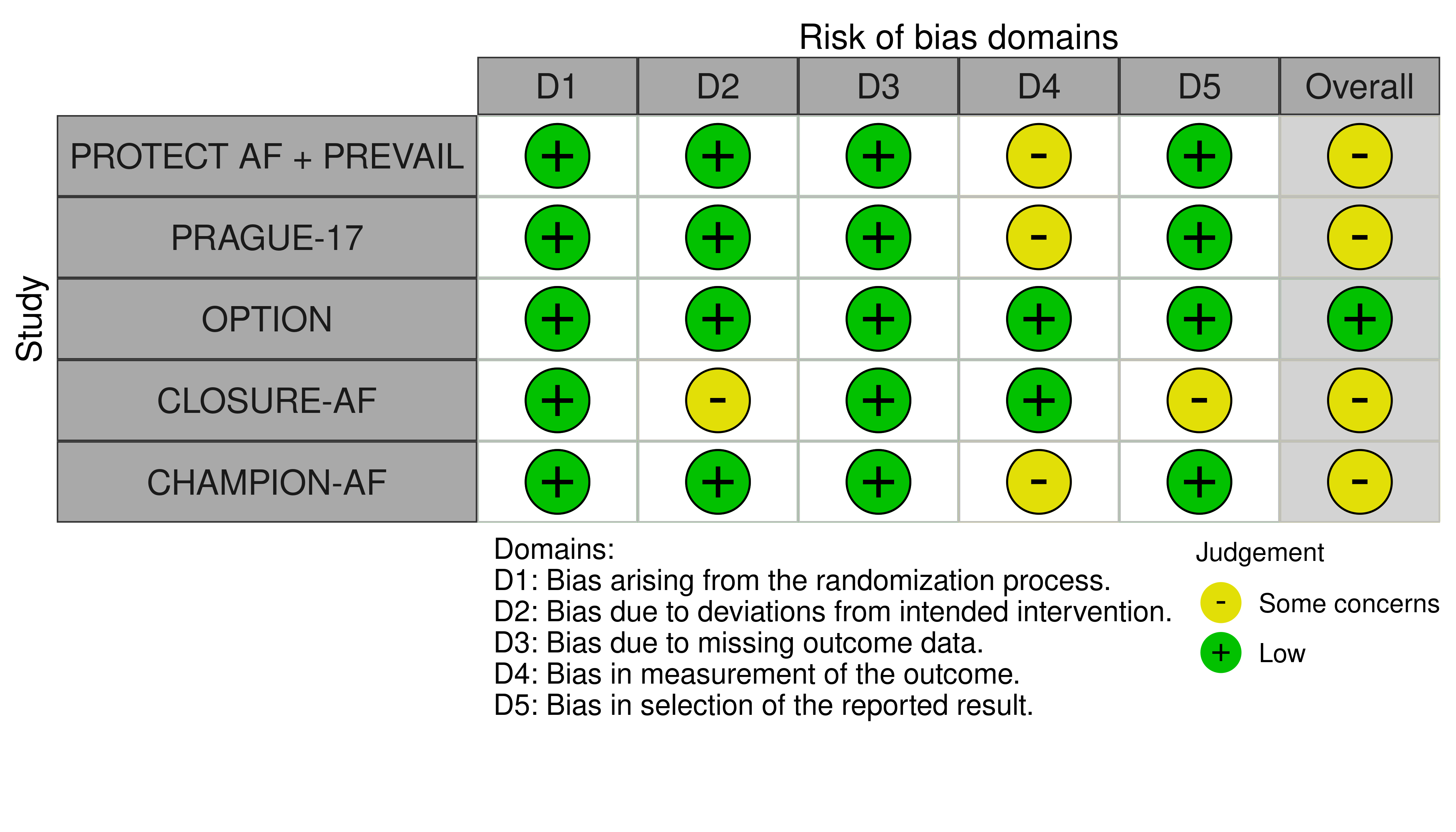


*Figure S2: Traffic light plot for stroke/systemic embolism*

## Clinically Relevant Bleeding


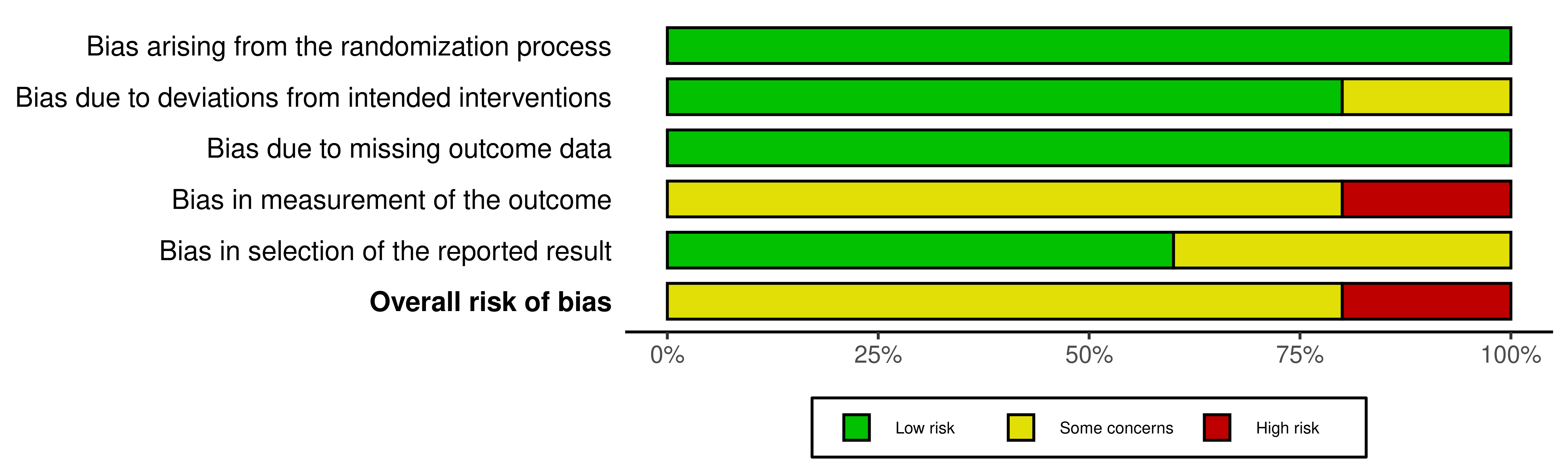


*Figure S3: Summary risk of bias plot for clinically relevant bleeding*


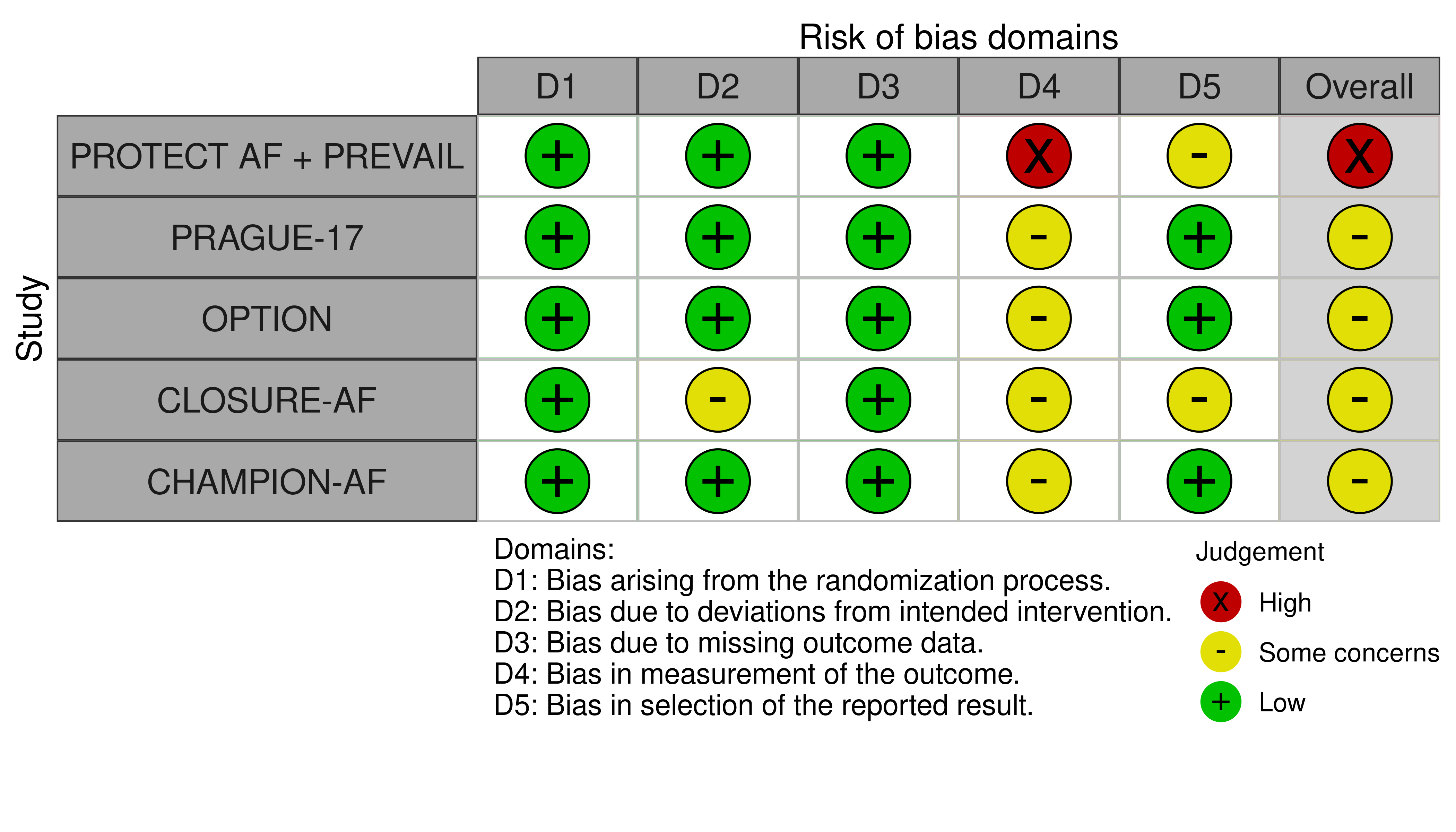


*Figure S4: Traffic light plot for clinically relevant bleeding*

Supplementary Appendix Methods S7 Publication Bias

## Funnel Plots

| Outcome | Estimate | SE | t_value | p_value |
| --- | --- | --- | --- | --- |
| SSE | -1.0717425 | 1.56372337 | -0.6853785 | 0.54231556 |
| CRB | 0.11294476 | 2.20205833 | 0.05129054 | 0.96231808 |


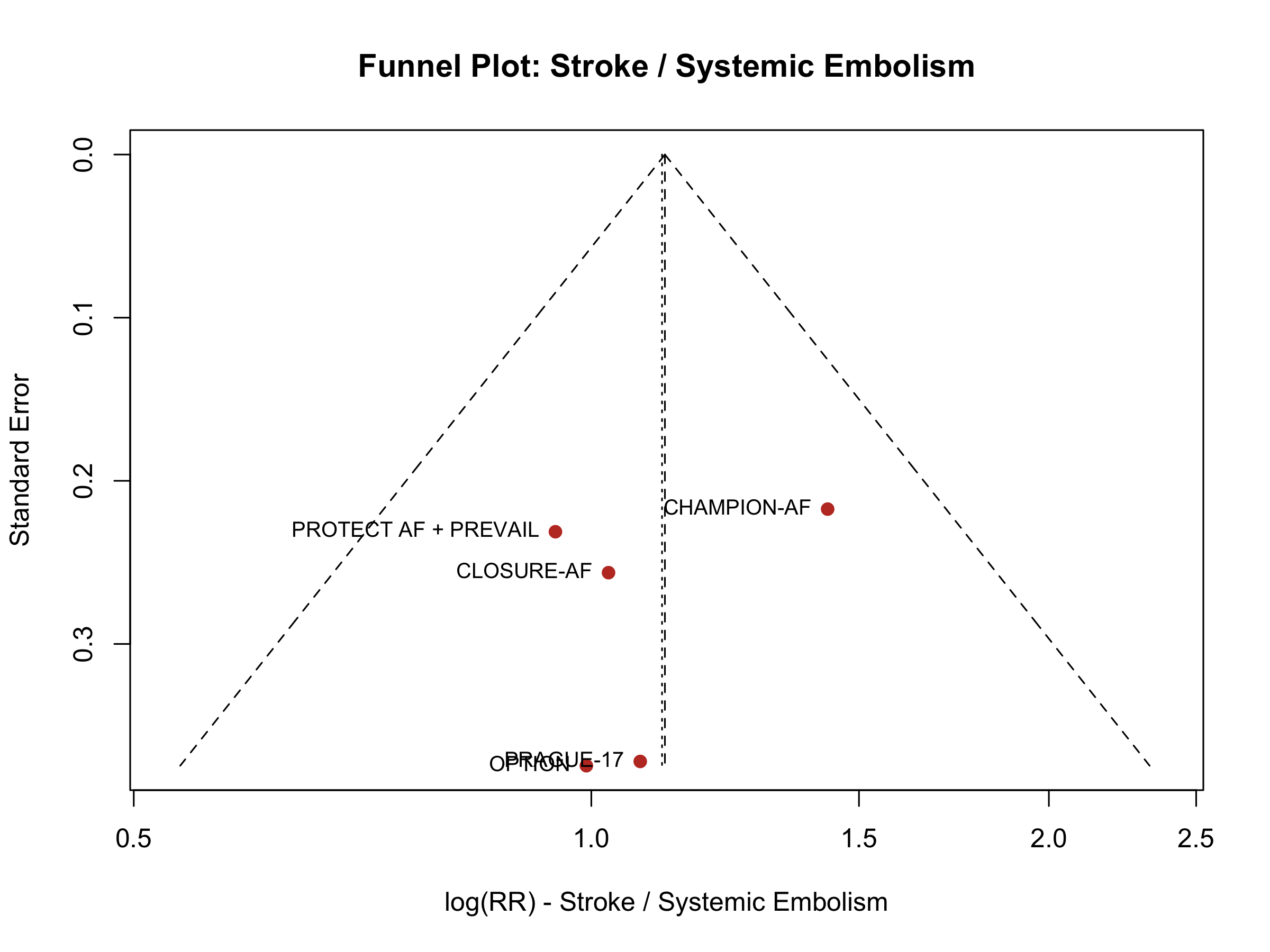


*Figure S5: Funnel plot showing no publication bias*


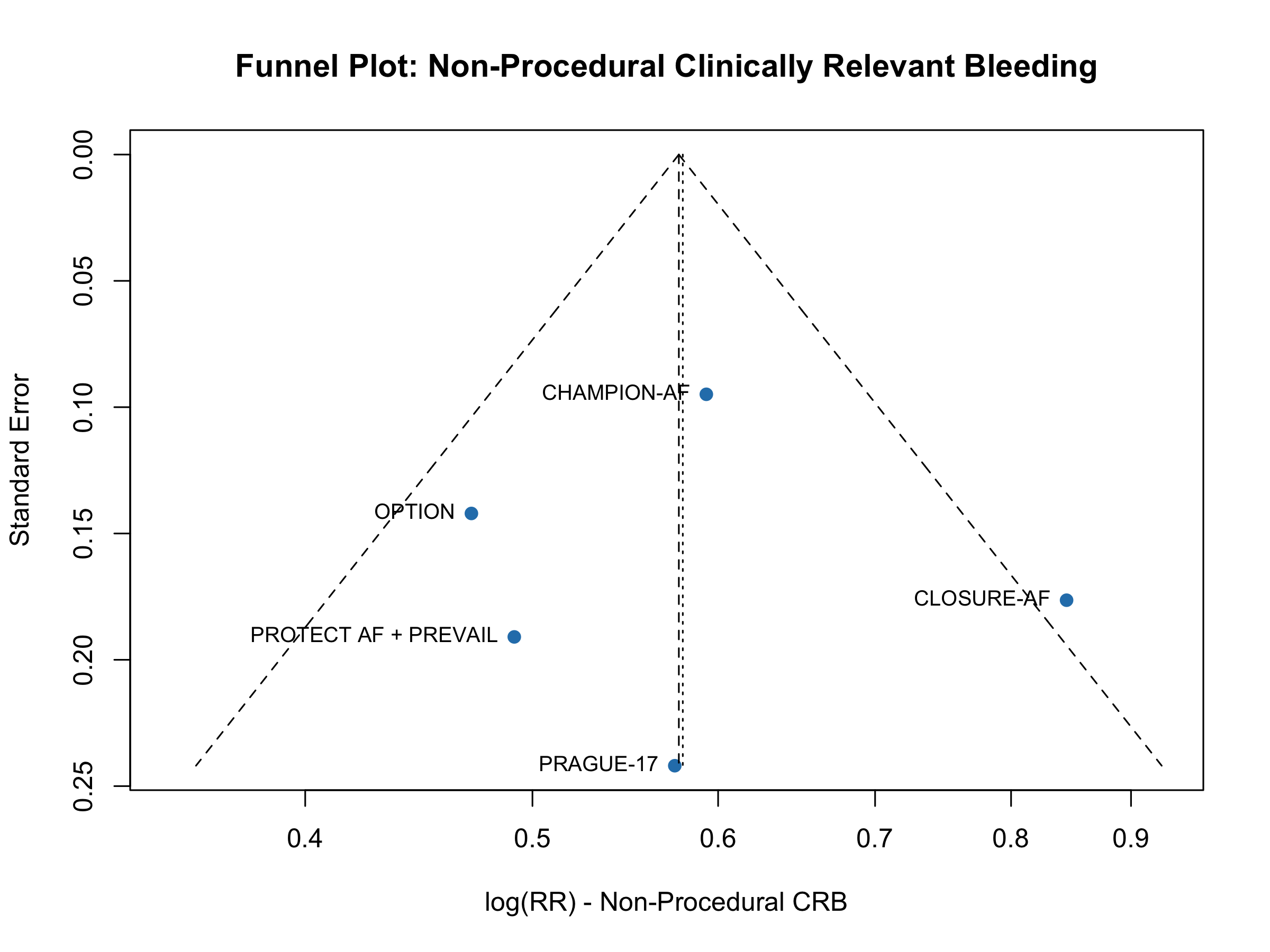


*Figure S6: Funnel plot showing no publication bias*

Supplementary Appendix Methods S8 Statistical analysis

The R code used was developed in accordance with the guidance from Harrer et al.^1^ the theoretical framework was laid according to the book "Bayesian Meta Analysis: A practical introduction"^2^

## Prior specifications

**Table S1. Prior specifications used across primary, sensitivity, and secondary outcome analyses**

| **Analysis** | **Prior category** | **Prior on μ (pooled log RR)** | **Prior on τ (between-study SD)** | **Rationale** |
| --- | --- | --- | --- | --- |
| Primary analysis | Weakly informative | Normal(0, 1) | Half-Cauchy(0, 0.5) | Broad range of plausible RR; standard weakly informative heterogeneity prior |
| Sensitivity 1 | Weakly informative | Normal(0, 1) | Half-Normal(0, 0.5) | Lighter tails; modestly more conservative for large heterogeneity |
| Sensitivity 2 | Weakly informative | Normal(0, 1) | Half-Student-t(df=3, 0, 0.5) | Intermediate tail behavior between Half-Cauchy and Half-Normal |
| Sensitivity 3 (Bartoš 2023)^3^ | Informative (empirical) | Student-t(df=2.28, 0, 0.26) | Inverse-Gamma(1.51, 4.35) | Derived from >50,000 Cochrane meta-analyses; realistic distribution of effect sizes and heterogeneity |
| Sensitivity 4 | Weakly informative | Normal(0, 0.5) | Half-Cauchy(0, 0.5) | Skeptical prior on effect; concentrates probability on smaller absolute log RR values |
| Sensitivity 5 | Weakly informative | Normal(0, 1.5) | Half-Cauchy(0, 0.5) | Diffuse prior on effect; permits large treatment effects |
| Sensitivity 6 (Informative) | Informative (prior MA) | SSE: Normal(−0.051, 0.173) CRB: Normal(−0.713, 0.108) | Half-Cauchy(0, 0.5) | Derived from prior MA of PROTECT AF, PREVAIL, PRAGUE-17, OPTION; Bayesian updating with CLOSURE-AF and CHAMPION-AF |
| Secondary outcomes | Weakly informative | Normal(0, 1) | Half-Cauchy(0, 0.5) | Consistent with primary analysis; exploratory interpretation |

**Table S2: Frequentist to Bayesian comparison**

| **Outcome** | **Method** | **Pooled RR** | **95% CI/CrI Lower** | **95% CI/CrI Upper** | **tau^2** | **I-squared (%)** | **Z / BF** | **P-value / P(RR<1)** |
| --- | --- | --- | --- | --- | --- | --- | --- | --- |
| SSE | Frequentist FE (MH) | 1.1179 | 0.8843 | 1.4133 | 0 | 0 | 0.932060427 | 0.351 |
| SSE | Frequentist RE (DL) | 1.1131 | 0.8798 | 1.4082 | 0 | 0 | 0.892872851 | 0.372 |
| SSE | Bayesian RE (HC 0.5) | 1.1142 | 0.8000 | 1.4977 |  |  | BF01=5.51 | 0.254 |
|  |  |  |  |  |  |  |  |  |
| CRB | Frequentist FE (MH) | 0.5773 | 0.5085 | 0.6554 | 0 | 0 | -8.490559022 | <0.001 |
| CRB | Frequentist RE (DL) | 0.5796 | 0.4771 | 0.7041 | 0.023237768 | 46.73833506 | -5.493642404 | <0.001 |
| CRB | Bayesian RE (HC 0.5) | 0.5910 | 0.4543 | 0.7755 |  |  | BF10=30.72 | 0.999 |
|  |  |  |  |  |  |  |  |  |
| CB | Frequentist FE (MH) | 0.8408 | 0.7514 | 0.9408 | 0 | 0 | -3.023603419 | 0.002 |
| CB | Frequentist RE (DL) | 0.8565 | 0.6062 | 1.2103 | 0.081616058 | 89.16960661 | -0.877953961 | 0.380 |
| CB | Bayesian RE (HC 0.5) | 0.9049 | 0.5173 | 1.5032 |  |  | BF10=0.29 | 0.761 |

## Convergence Diagnostics

**Table S3: MCMC Convergence Diagnostics**

| **Outcome** | **Prior** | **R-hat (mu)** | **R-hat (tau)** | **Bulk ESS (mu)** | **Bulk ESS (tau)** | **Tail ESS (mu)** | **Tail ESS (tau)** | **Status** |
| --- | --- | --- | --- | --- | --- | --- | --- | --- |
| SSE | mu~N(0,1), tau~Half-Cauchy(0,0.5) [Primary] | 1.001 | 1.001 | 7568 | 6538 | 6254 | 7162 | PASS |
| SSE | mu~N(0,1), tau~Half-Normal(0,0.5) | 1.000 | 1.000 | 9913 | 7386 | 8818 | 7854 | PASS |
| SSE | mu~N(0,1), tau~Half-Student-t(3,0,0.5) | 1.000 | 1.000 | 9972 | 7130 | 8893 | 8400 | PASS |
| SSE | Empirical [Bartos 2023] | 1.001 | 1.001 | 5948 | 5337 | 7310 | 5705 | PASS |
| SSE | mu~N(0,0.5), tau~Half-Cauchy(0,0.5) [Skeptical] | 1.001 | 1.000 | 10984 | 7574 | 9084 | 7353 | PASS |
| SSE | mu~N(0,1.5), tau~Half-Cauchy(0,0.5) [Diffuse] | 1.000 | 1.000 | 8868 | 7393 | 7628 | 7962 | PASS |
| SSE | Informative: mu~N(-0.051,0.173) [Prior MA] | 1.000 | 1.001 | 13136 | 7241 | 10113 | 7285 | PASS |
| CRB | mu~N(0,1), tau~Half-Cauchy(0,0.5) [Primary] | 1.000 | 1.001 | 6072 | 4320 | 5798 | 5990 | PASS |
| CRB | mu~N(0,1), tau~Half-Normal(0,0.5) | 1.000 | 1.001 | 4934 | 4122 | 5153 | 6485 | PASS |
| CRB | mu~N(0,1), tau~Half-Student-t(3,0,0.5) | 1.001 | 1.000 | 4711 | 4087 | 4804 | 5963 | PASS |
| CRB | Empirical [Bartos 2023] | 1.001 | 1.001 | 3486 | 4054 | 4079 | 4600 | PASS |
| CRB | mu~N(0,0.5), tau~Half-Cauchy(0,0.5) [Skeptical] | 1.001 | 1.001 | 5594 | 4113 | 4491 | 5577 | PASS |
| CRB | mu~N(0,1.5), tau~Half-Cauchy(0,0.5) [Diffuse] | 1.001 | 1.002 | 5275 | 3779 | 4871 | 6345 | PASS |
| CRB | Informative: mu~N(-0.713,0.108) [Prior MA] | 1.001 | 1.001 | 7662 | 4914 | 7668 | 6415 | PASS |
| Clinical Benefit | mu~N(0,1), tau~Half-Cauchy(0,0.5) [Primary] | 1.002 | 1.001 | 4054 | 3436 | 4569 | 5489 | PASS |
| Clinical Benefit | mu~N(0,1), tau~Half-Normal(0,0.5) | 1.000 | 1.001 | 4511 | 4110 | 4800 | 6927 | PASS |
| Clinical Benefit | mu~N(0,1), tau~Half-Student-t(3,0,0.5) | 1.001 | 1.001 | 4307 | 3838 | 4270 | 5938 | PASS |
| Clinical Benefit | Empirical [Bartos 2023] | 1.001 | 1.000 | 4674 | 3851 | 5026 | 4779 | PASS |
| Clinical Benefit | mu~N(0,0.5), tau~Half-Cauchy(0,0.5) [Skeptical] | 1.001 | 1.001 | 4466 | 4158 | 5188 | 5615 | PASS |
| Clinical Benefit | mu~N(0,1.5), tau~Half-Cauchy(0,0.5) [Diffuse] | 1.001 | 1.000 | 4048 | 3890 | 4726 | 4918 | PASS |
| Ischemic Stroke | Half-Cauchy(0,0.5) | 1.001 | 1.000 | 10198 | 7298 | 6881 | 7842 | PASS |
| Hemorrhagic Stroke | Half-Cauchy(0,0.5) | 1.000 | 1.001 | 9911 | 6879 | 8776 | 7304 | PASS |
| All-Cause Bleeding | Half-Cauchy(0,0.5) | 1.000 | 1.001 | 7309 | 5615 | 6224 | 6946 | PASS |
| CV/Unexplained Death | Half-Cauchy(0,0.5) | 1.000 | 1.001 | 5752 | 4925 | 6428 | 5725 | PASS |

*Criteria: R-hat < 1.1, ESS > 400. MCMC: 4 chains via brms/Stan (NUTS sampler), 10,000 iterations, 5,000 warmup. Based on the Vehtari et al and the previous Gelman and Rubin potential scale reduction factor recommendations*^4^

## Trace Plots


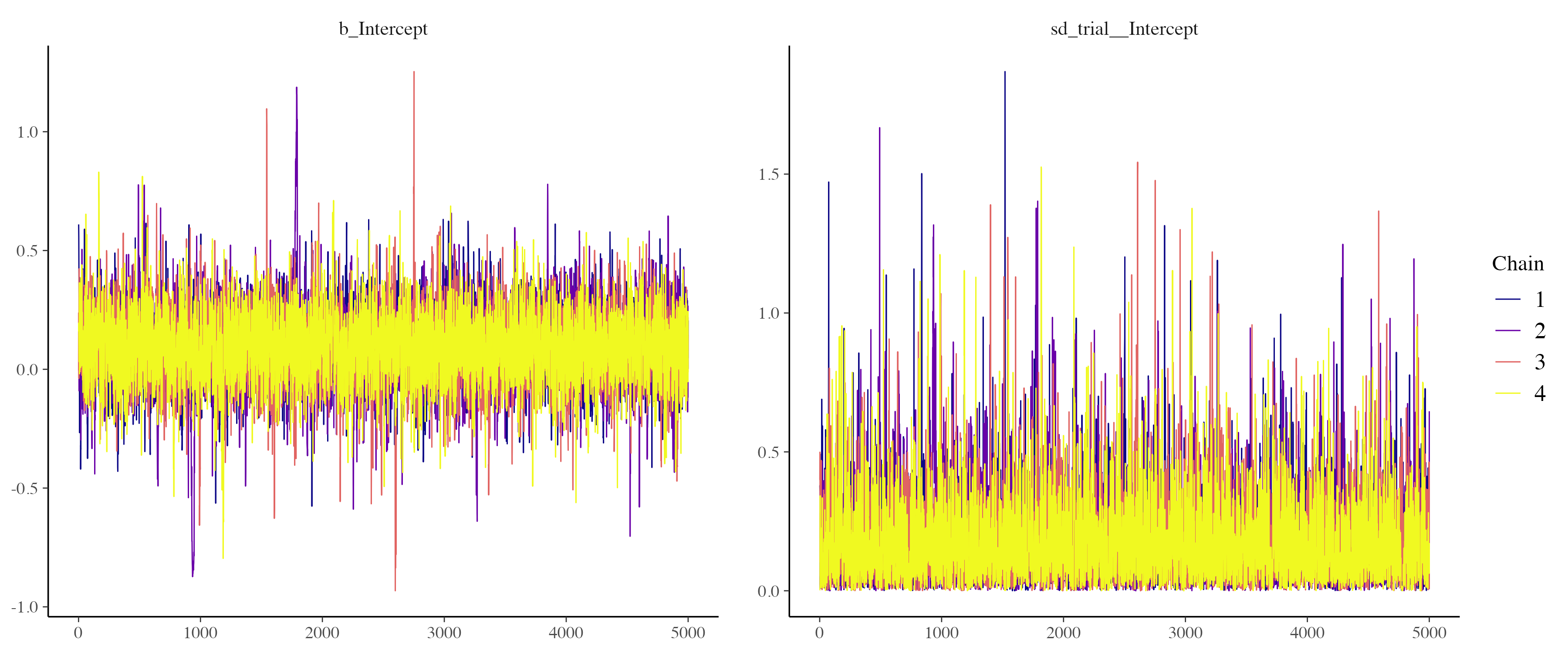


*Figure S5: Trace plot for stroke/systemic embolism showing model convergence*


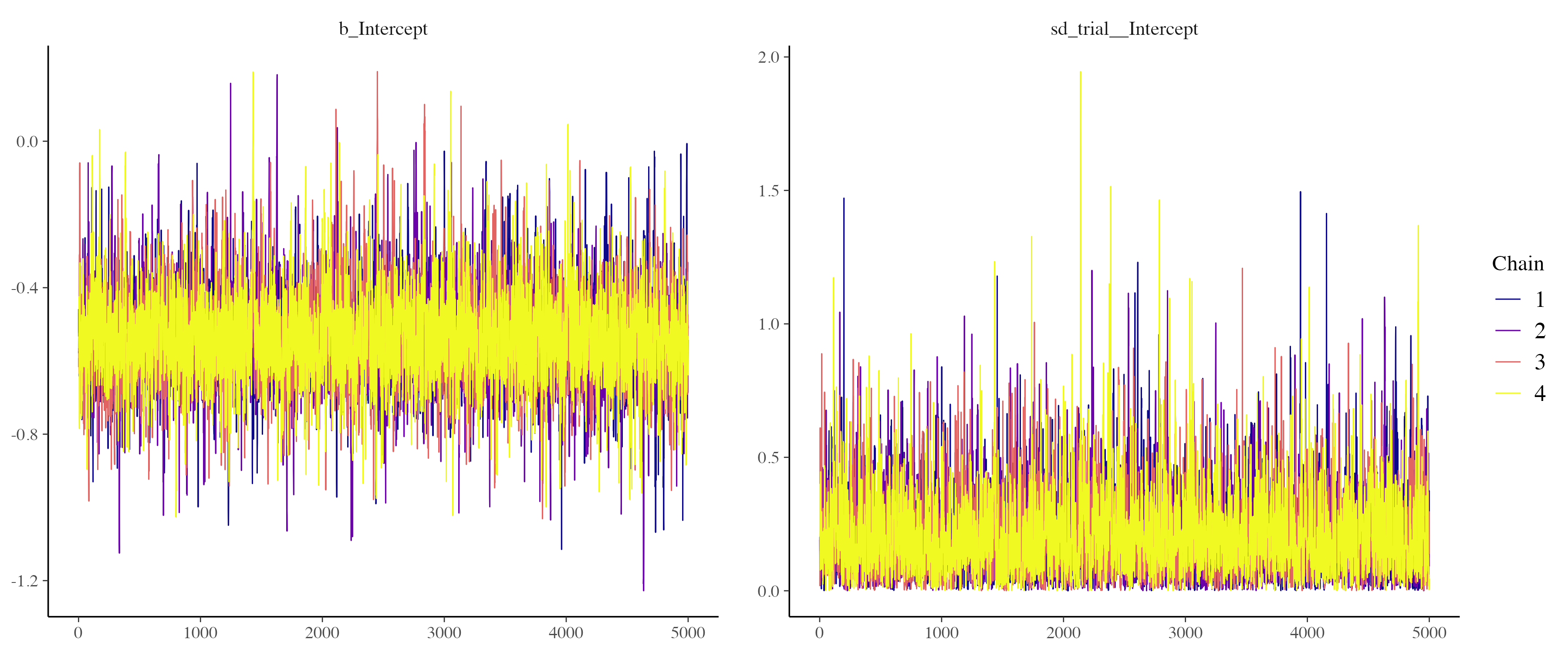


*Figure S6: Trace plot for clinically relevant bleeding showing model convergence*


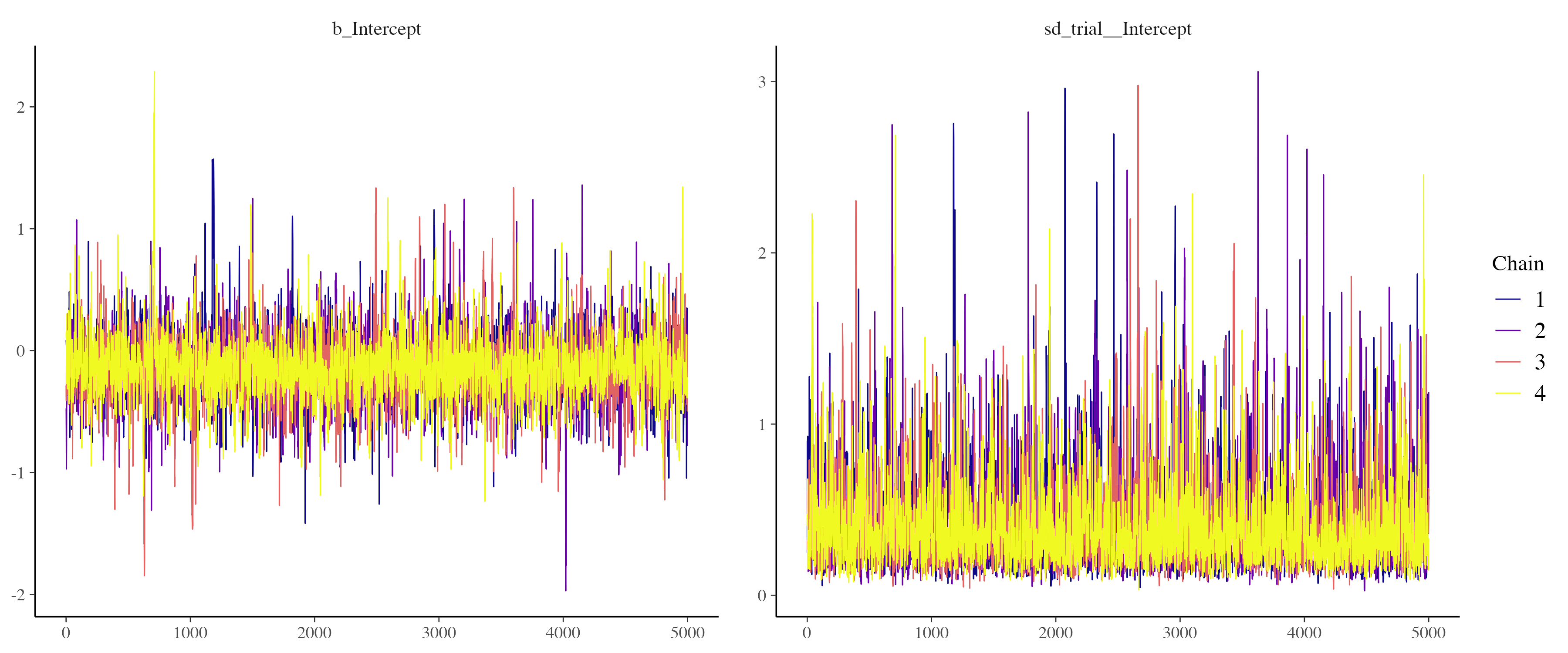


*Figure S7: Trace plot for clinical benefit showing model convergence*

## Posterior Predictive Check Plots


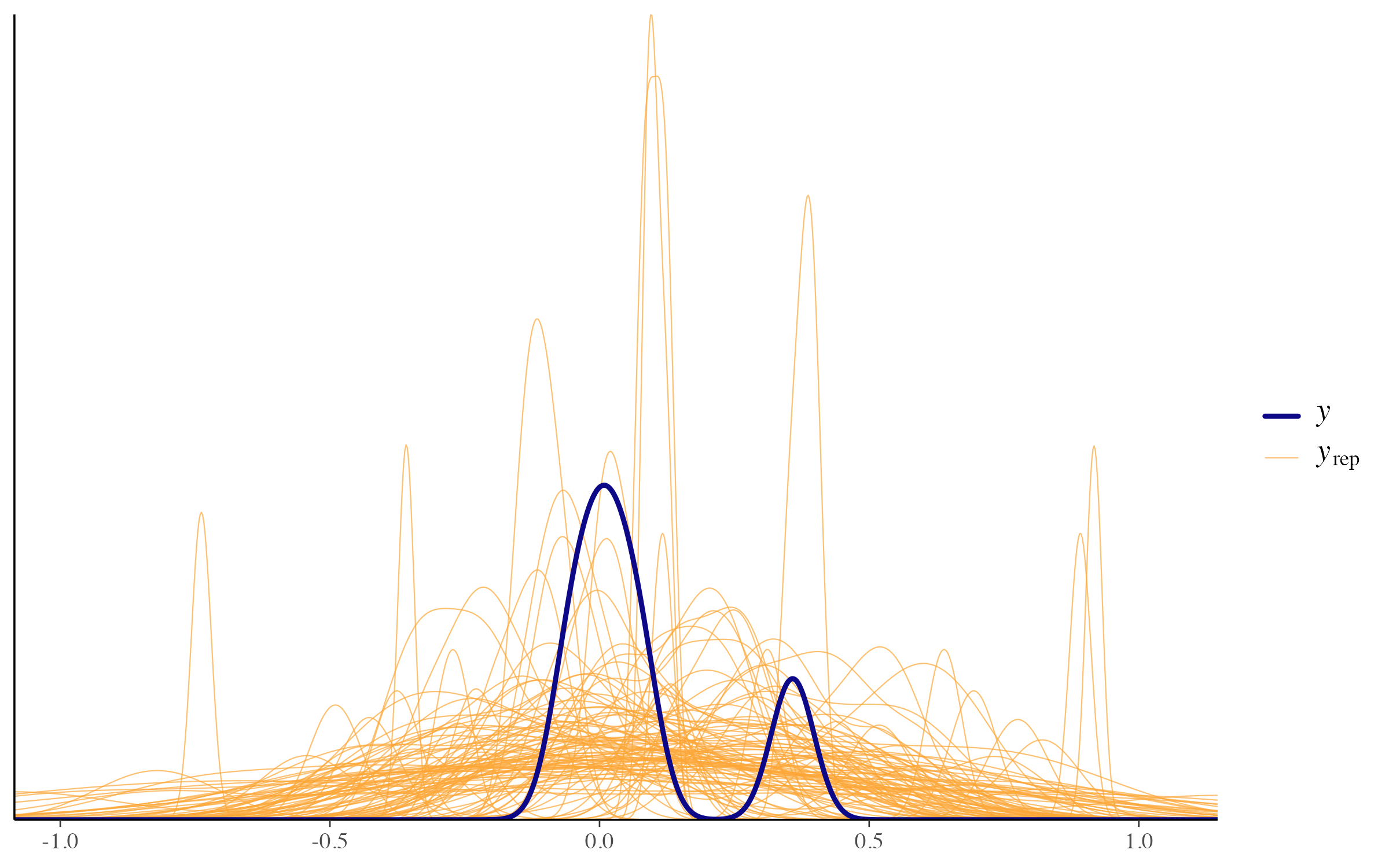


*Figure S8: Posterior predictive check for stroke or systemic embolism showing good fit with one divergent series*


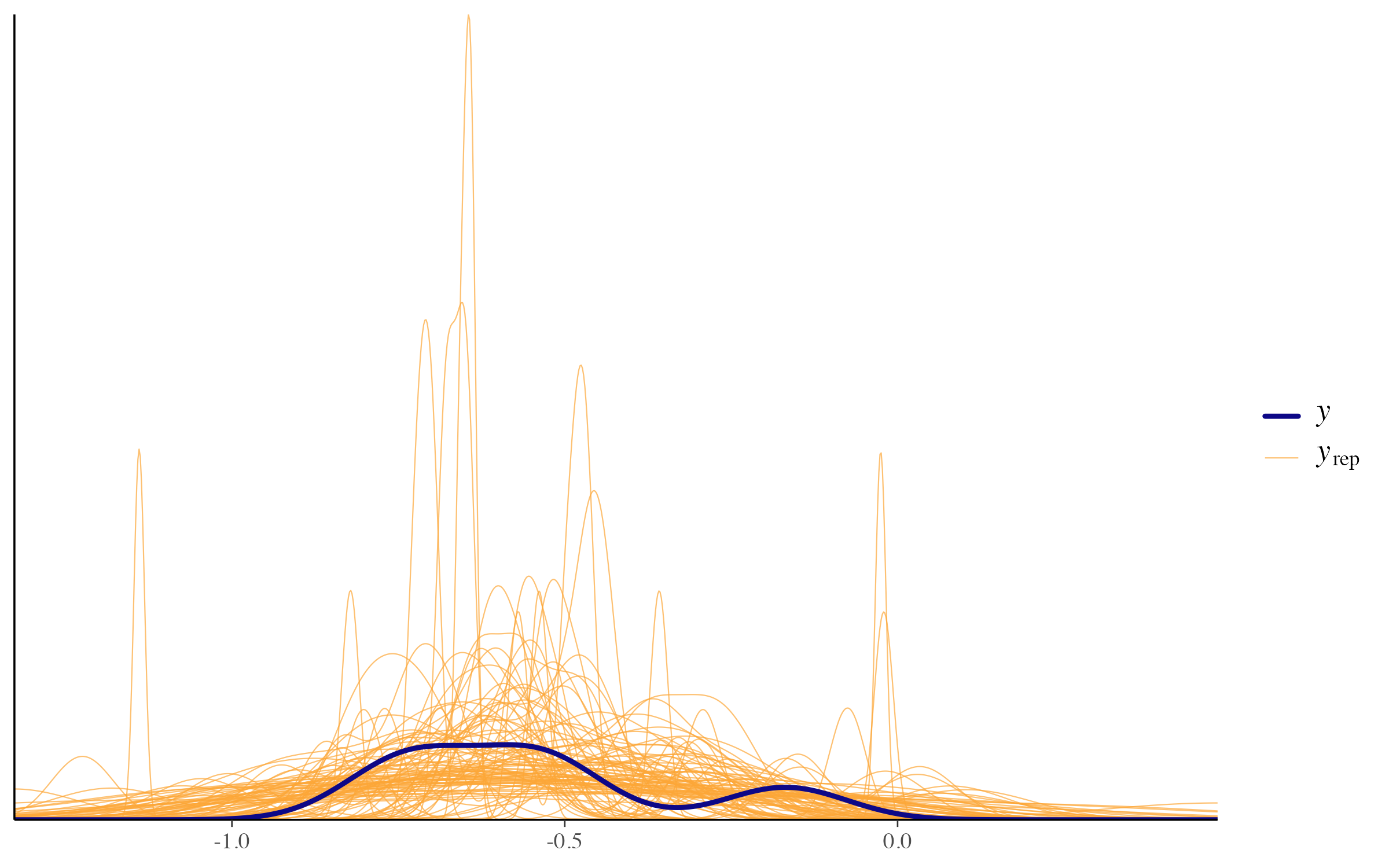
*Figure S9: Posterior predictive check for clinically relevant bleeding showing good fit with one divergent series*


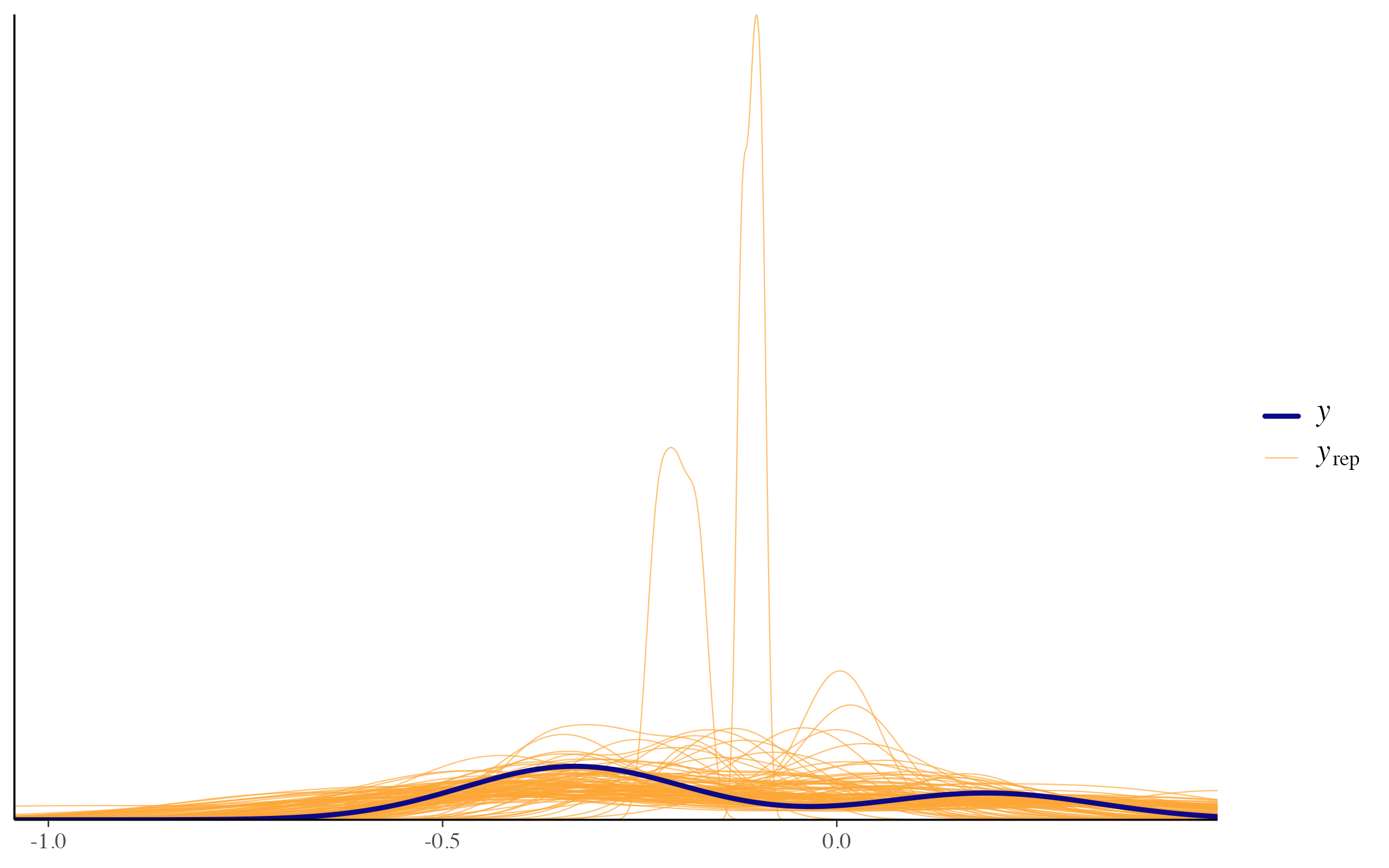


*Figure S10: Posterior predictive check for clinical benefit showing good fit with two divergent series*

Supplementary Appendix Results S9 Sensitivity analysis

**Table S4: Prior Sensitivity Analysis: Results across different prior specifications**

| **Outcome** | **Prior Specification** | **Pooled RR** | **95% CrI Lower** | **95% CrI Upper** | **tau (Mean)** | **tau 95% CrI Lower** | **tau 95% CrI Upper** | **P(RR<1)** | **R-hat (mu)** | **Interpretation** |
| --- | --- | --- | --- | --- | --- | --- | --- | --- | --- | --- |
| Stroke / Systemic Embolism | μ~N(0,1), τ~Half-Cauchy(0,0.5) [Primary] | 1.114 | 0.800 | 1.498 | 0.178 | 0.006 | 0.593 | 0.254 | 1.001 | No difference |
| Stroke / Systemic Embolism | μ~N(0,1), τ~Half-Normal(0,0.5) | 1.115 | 0.802 | 1.499 | 0.181 | 0.007 | 0.565 | 0.251 | 1.000 | No difference |
| Stroke / Systemic Embolism | μ~N(0,1), τ~Half-Student-t(3,0,0.5) | 1.116 | 0.804 | 1.490 | 0.176 | 0.007 | 0.564 | 0.246 | 1.000 | No difference |
| Stroke / Systemic Embolism | Empirical: μ~St-t(2.28,0,0.26), τ~InvGamma(1.51,4.35) [Bartos 2023] | 1.061 | 0.612 | 1.718 | 0.957 | 0.434 | 2.098 | 0.451 | 1.001 | No difference |
| Stroke / Systemic Embolism | μ~N(0,0.5), τ~Half-Cauchy(0,0.5) [Skeptical] | 1.106 | 0.811 | 1.451 | 0.172 | 0.007 | 0.551 | 0.259 | 1.000 | No difference |
| Stroke / Systemic Embolism | μ~N(0,1.5), τ~Half-Cauchy(0,0.5) [Diffuse] | 1.116 | 0.793 | 1.500 | 0.179 | 0.007 | 0.592 | 0.253 | 1.000 | No difference |
| Stroke / Systemic Embolism | Informative: μ~N(-0.051,0.173), τ~Half-Cauchy(0,0.5) [Prior MA] | 1.046 | 0.824 | 1.295 | 0.166 | 0.006 | 0.533 | 0.357 | 1.000 | No difference |
| Non-Procedural CRB | μ~N(0,1), τ~Half-Cauchy(0,0.5) [Primary] | 0.591 | 0.454 | 0.776 | 0.202 | 0.010 | 0.577 | 0.999 | 1.000 | Favors LAAO |
| Non-Procedural CRB | μ~N(0,1), τ~Half-Normal(0,0.5) | 0.591 | 0.453 | 0.778 | 0.212 | 0.012 | 0.585 | 0.998 | 1.000 | Favors LAAO |
| Non-Procedural CRB | μ~N(0,1), τ~Half-Student-t(3,0,0.5) | 0.591 | 0.453 | 0.775 | 0.206 | 0.011 | 0.583 | 0.998 | 1.001 | Favors LAAO |
| Non-Procedural CRB | Empirical: μ~St-t(2.28,0,0.26), τ~InvGamma(1.51,4.35) [Bartos 2023] | 0.847 | 0.437 | 1.405 | 1.019 | 0.459 | 2.162 | 0.774 | 1.000 | No difference |
| Non-Procedural CRB | μ~N(0,0.5), τ~Half-Cauchy(0,0.5) [Skeptical] | 0.605 | 0.478 | 0.803 | 0.203 | 0.011 | 0.585 | 0.996 | 1.001 | Favors LAAO |
| Non-Procedural CRB | μ~N(0,1.5), τ~Half-Cauchy(0,0.5) [Diffuse] | 0.589 | 0.451 | 0.771 | 0.206 | 0.011 | 0.597 | 0.997 | 1.001 | Favors LAAO |
| Non-Procedural CRB | Informative: μ~N(-0.713,0.108), τ~Half-Cauchy(0,0.5) [Prior MA] | 0.534 | 0.448 | 0.618 | 0.202 | 0.012 | 0.551 | 1.000 | 1.001 | Favors LAAO |
| Clinical Benefit Composite | μ~N(0,1), τ~Half-Cauchy(0,0.5) [Primary] | 0.905 | 0.517 | 1.503 | 0.413 | 0.126 | 1.125 | 0.761 | 1.000 | No difference |
| Clinical Benefit Composite | μ~N(0,1), τ~Half-Normal(0,0.5) | 0.889 | 0.510 | 1.434 | 0.387 | 0.126 | 0.901 | 0.772 | 1.001 | No difference |
| Clinical Benefit Composite | μ~N(0,1), τ~Half-Student-t(3,0,0.5) | 0.891 | 0.510 | 1.464 | 0.403 | 0.122 | 1.021 | 0.775 | 1.001 | No difference |
| Clinical Benefit Composite | Empirical: μ~St-t(2.28,0,0.26), τ~InvGamma(1.51,4.35) [Bartos 2023] | 1.014 | 0.497 | 1.827 | 1.363 | 0.515 | 3.461 | 0.552 | 1.001 | No difference |
| Clinical Benefit Composite | μ~N(0,0.5), τ~Half-Cauchy(0,0.5) [Skeptical] | 0.901 | 0.575 | 1.395 | 0.399 | 0.124 | 1.056 | 0.759 | 1.000 | No difference |
| Clinical Benefit Composite | μ~N(0,1.5), τ~Half-Cauchy(0,0.5) [Diffuse] | 0.898 | 0.483 | 1.534 | 0.428 | 0.127 | 1.220 | 0.760 | 1.000 | No difference |

Supplementary Appendix Results S10 Additional results

## Study level data

| Primary Efficacy: Stroke or Systemic Embolism (SSE) | | |  |  |  |  |  |  |  |
| --- | --- | --- | --- | --- | --- | --- | --- | --- | --- |
| Trial | Events LAAO | N LAAO | Events OAC | N OAC | Risk Ratio | log(RR) | SE(log RR) | 95% CI Lower | 95% CI Upper |
| PROTECT AF + PREVAIL | 49 | 732 | 27 | 382 | 0.9471 | -0.0544 | 0.2312 | 0.6020 | 1.4900 |
| PRAGUE-17 | 14 | 201 | 13 | 201 | 1.0769 | 0.0741 | 0.3720 | 0.5194 | 2.2328 |
| OPTION | 14 | 803 | 14 | 797 | 0.9925 | -0.0075 | 0.3746 | 0.4763 | 2.0685 |
| CLOSURE-AF | 29 | 446 | 28 | 442 | 1.0264 | 0.0261 | 0.2563 | 0.6211 | 1.6963 |
| CHAMPION-AF | 50 | 1499 | 35 | 1501 | 1.4305 | 0.3580 | 0.2173 | 0.9343 | 2.1902 |
|  |  |  |  |  |  |  |  |  |  |
| Primary Safety: Non-Procedural CRB | |  |  |  |  |  |  |  |  |
| Trial | Events LAAO | N LAAO | Events OAC | N OAC | Risk Ratio | log(RR) | SE(log RR) | 95% CI Lower | 95% CI Upper |
| PROTECT AF + PREVAIL | 48 | 732 | 51 | 382 | 0.4912 | -0.7110 | 0.1909 | 0.3378 | 0.7141 |
| PRAGUE-17 | 23 | 201 | 40 | 201 | 0.5750 | -0.5534 | 0.2419 | 0.3579 | 0.9238 |
| OPTION | 65 | 803 | 137 | 797 | 0.4709 | -0.7531 | 0.1421 | 0.3565 | 0.6221 |
| CLOSURE-AF | 52 | 446 | 61 | 442 | 0.8448 | -0.1686 | 0.1764 | 0.5979 | 1.1938 |
| CHAMPION-AF | 154 | 1499 | 260 | 1501 | 0.5931 | -0.5224 | 0.0949 | 0.4924 | 0.7143 |
|  |  |  |  |  |  |  |  |  |  |
| Clinical Benefit Composite |  |  |  |  |  |  |  |  |  |
| Trial | Events LAAO | N LAAO | Events OAC | N OAC | Risk Ratio | log(RR) | SE(log RR) | 95% CI Lower | 95% CI Upper |
| CLOSURE-AF | 155 | 446 | 127 | 442 | 1.2095 | 0.1902 | 0.0991 | 0.9960 | 1.4688 |
| PRAGUE-17 | 58 | 201 | 81 | 201 | 0.7160 | -0.3340 | 0.1401 | 0.5441 | 0.9424 |
| CHAMPION-AF | 215 | 1499 | 300 | 1501 | 0.7176 | -0.3318 | 0.0816 | 0.6116 | 0.8420 |
|  |  |  |  |  |  |  |  |  |  |
| Ischemic Stroke |  |  |  |  |  |  |  |  |  |
| Trial | Events LAAO | N LAAO | Events OAC | N OAC | Risk Ratio | log(RR) | SE(log RR) | 95% CI Lower | 95% CI Upper |
| CHAMPION-AF | 45 | 1499 | 27 | 1501 | 1.6689 | 0.5122 | 0.2407 | 1.0413 | 2.6748 |
| CLOSURE-AF | 18 | 446 | 15 | 442 | 1.1892 | 0.1733 | 0.3431 | 0.6070 | 2.3298 |
| OPTION | 9 | 803 | 10 | 797 | 0.8933 | -0.1129 | 0.4567 | 0.3649 | 2.1866 |
| PRAGUE-17 | 13 | 201 | 10 | 201 | 1.3000 | 0.2624 | 0.4086 | 0.5836 | 2.8958 |
| PROTECT AF + PREVAIL | 45 | 732 | 14 | 382 | 1.6774 | 0.5172 | 0.2994 | 0.9327 | 3.0167 |
|  |  |  |  |  |  |  |  |  |  |
| Hemorrhagic Stroke |  |  |  |  |  |  |  |  |  |
| Trial | Events LAAO | N LAAO | Events OAC | N OAC | Risk Ratio | log(RR) | SE(log RR) | 95% CI Lower | 95% CI Upper |
| CHAMPION-AF | 5 | 1499 | 5 | 1501 | 1.0013 | 0.0013 | 0.6314 | 0.2905 | 3.4517 |
| CLOSURE-AF | 10 | 446 | 13 | 442 | 0.7623 | -0.2714 | 0.4152 | 0.3378 | 1.7203 |
| OPTION | 3 | 803 | 3 | 797 | 0.9925 | -0.0075 | 0.8150 | 0.2009 | 4.9029 |
| PRAGUE-17 | 1 | 201 | 2 | 201 | 0.5000 | -0.6931 | 1.2207 | 0.0457 | 5.4705 |
| PROTECT AF + PREVAIL | 5 | 732 | 13 | 382 | 0.2007 | -1.6059 | 0.5224 | 0.0721 | 0.5588 |
|  |  |  |  |  |  |  |  |  |  |
| All-Cause Bleeding |  |  |  |  |  |  |  |  |  |
| Trial | Events LAAO | N LAAO | Events OAC | N OAC | Risk Ratio | log(RR) | SE(log RR) | 95% CI Lower | 95% CI Upper |
| CHAMPION-AF | 83 | 1499 | 87 | 1501 | 0.9553 | -0.0457 | 0.1490 | 0.7133 | 1.2794 |
| CLOSURE-AF | 70 | 446 | 61 | 442 | 1.1372 | 0.1286 | 0.1618 | 0.8282 | 1.5616 |
| OPTION | 30 | 803 | 38 | 797 | 0.7836 | -0.2439 | 0.2391 | 0.4904 | 1.2519 |
| PRAGUE-17 | 29 | 201 | 40 | 201 | 0.7250 | -0.3216 | 0.2226 | 0.4687 | 1.1215 |
| PROTECT AF + PREVAIL | 85 | 732 | 50 | 382 | 0.8872 | -0.1197 | 0.1667 | 0.6399 | 1.2299 |

**Subgroup based on Newer device generation- secondary outcomes**

|  | median | lower | upper | psup |
| --- | --- | --- | --- | --- |
| Ischemic_Stroke | 1.30950684 | 0.6876557 | 2.19480463 | 0.15765 |
| Hemorrhagic_Stroke | 0.87676584 | 0.4272672 | 1.93763456 | 0.63935 |
| All_Cause_Bleeding | 0.97503957 | 0.66043538 | 1.41431299 | 0.57185 |
| CV_Unexpl_Death | 1.12847295 | 0.71925806 | 1.65238519 | 0.23955 |

**Subgroup analysis based on DOAC-era studies**

|  | median | lower | upper | psup |
| --- | --- | --- | --- | --- |
| Ischemic_Stroke | 1.31359912 | 0.81431454 | 1.99664042 | 0.10675 |
| Hemorrhagic_Stroke | 0.84285638 | 0.41683871 | 1.71223599 | 0.688 |
| All_Cause_Bleeding | 0.9215993 | 0.66070525 | 1.23665419 | 0.74675 |
| CV_Unexpl_Death | 1.02342418 | 0.68519778 | 1.44226452 | 0.44125 |

## Study Level Plots


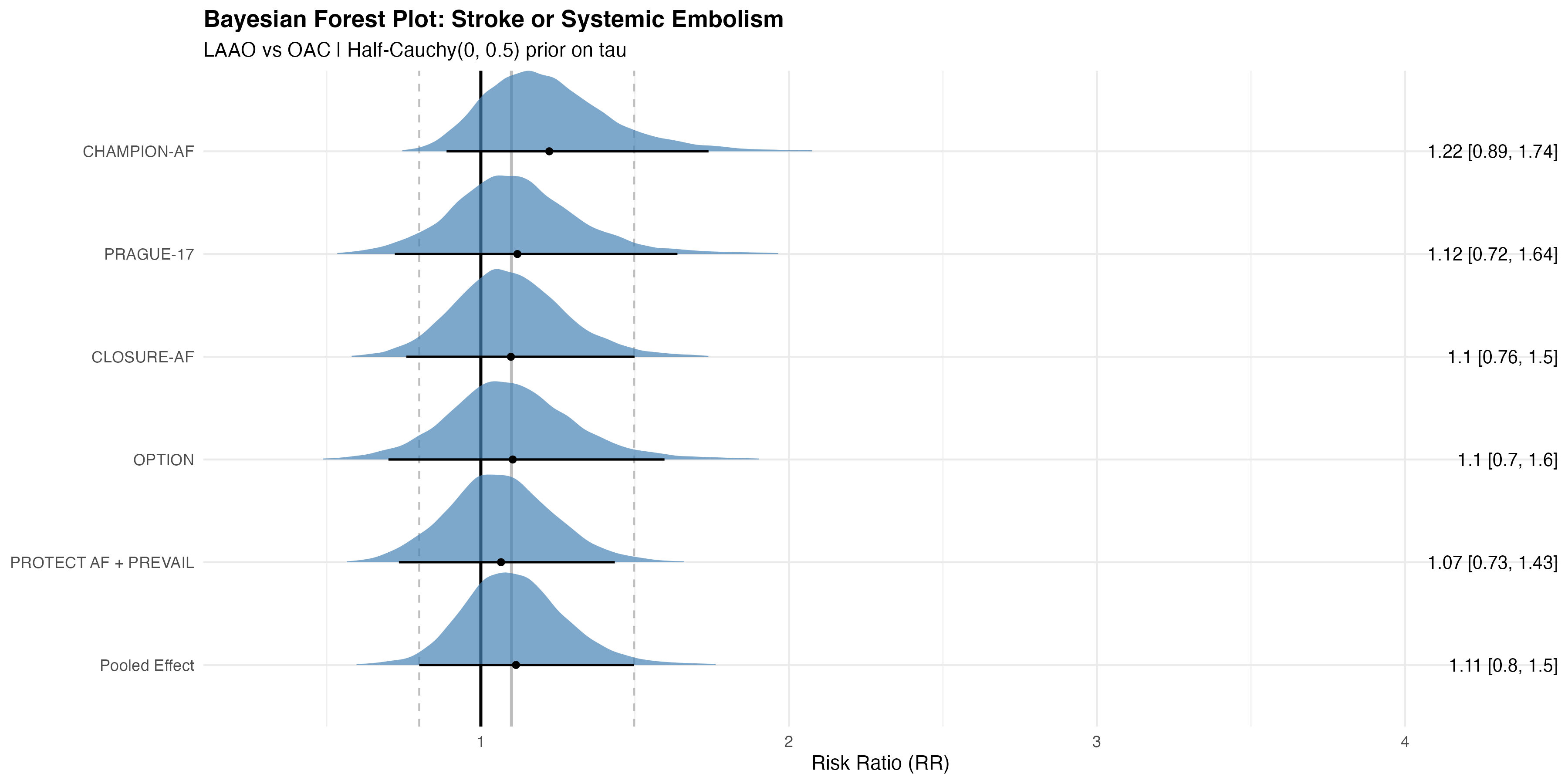


*Figure S11: Forest Plot for Stroke and Systemic Embolism*


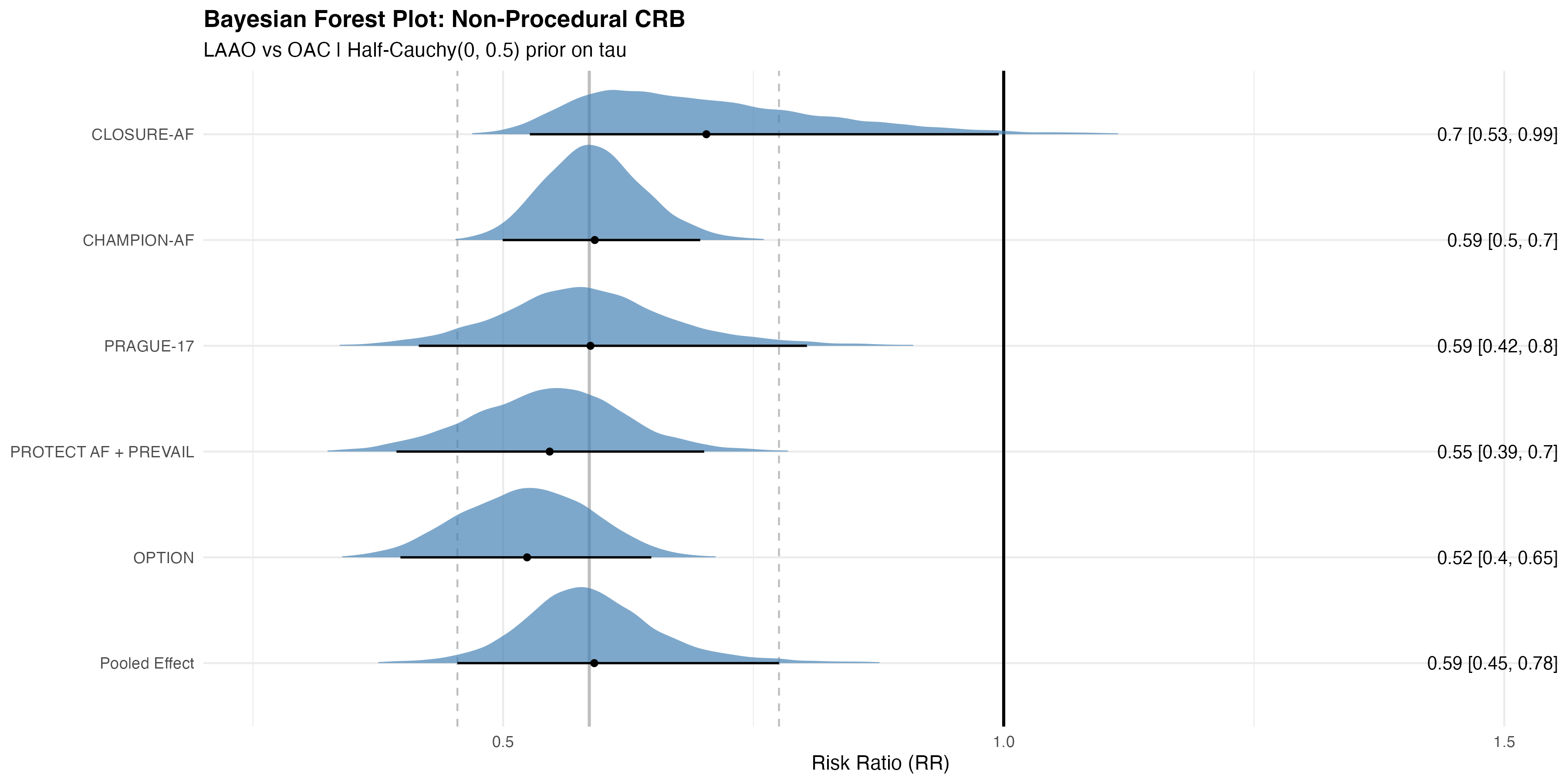


*Figure S12: Forest Plot for Clinically Relevant Bleeding*


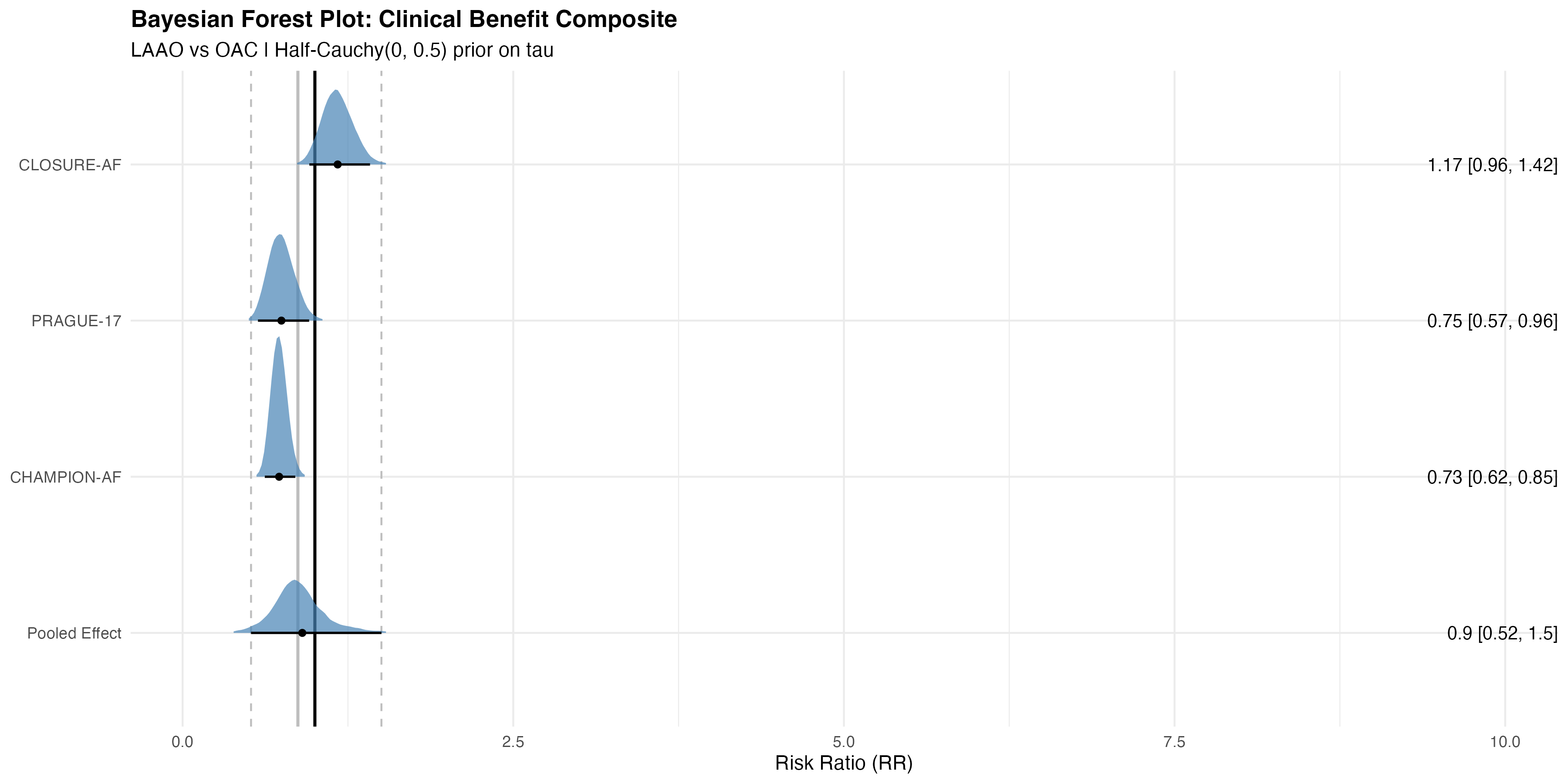


*Figure S13: Forest Plot for Overall Clinical Benefit*

**Sensitivity analysis for heterogeneity assessment for PROTECT and PREVIAL:**

On request of reviewers a post hoc analysis for stroke or systemic embolism outcome was done to assess consistency of between-study Heterogeneity by taking protect and prevail as separate trial units.

| Analysis | Median_RR | CrI_Lower | CrI_Upper | P_RR_gt_1 | tau_median |
| --- | --- | --- | --- | --- | --- |
| Primary (5 units, PROTECT+PREVAIL combined) | 1.101810021 | 0.800049253 | 1.497715021 | 0.7456 | 0.138738079 |
| Sensitivity (6 trials, PROTECT & PREVAIL separate) | 1.101428726 | 0.813632147 | 1.497464426 | 0.7479 | 0.148992124 |

Supplementary Appendix Results S11 References

1. Harrer M, Cuijpers P, Furukawa TA, Ebert DD. *Doing Meta-Analysis with R: A Hands-on Guide*. First edition. CRC Press, Taylor & Francis Group; 2022. doi:10.1201/9781003107347

2. Grant R, Di Tanna GL. *Bayesian Meta-Analysis: A Practical Introduction*. 1st ed. Chapman and Hall/CRC; 2025. doi:10.1201/9781003375821

3. Bartoš F, Otte WM, Gronau QF, Timmers B, Ly A, Wagenmakers EJ. Empirical prior distributions for Bayesian meta-analyses of binary and time to event outcomes. *arXiv*. Preprint posted online 2023. doi:10.48550/ARXIV.2306.11468

4. Vehtari A, Gelman A, Simpson D, Carpenter B, Bürkner PC. Rank-Normalization, Folding, and Localization: An Improved Rˆ for Assessing Convergence of MCMC (with Discussion). *Bayesian Anal*. 2021;16(2). doi:10.1214/20-BA1221
